# Left Write Hook: trial protocol for a community-based type II hybrid effectiveness-implementation cluster randomised controlled trial of a boxing and writing-based intervention for adult survivors of child sexual abuse and gender-based violence in Australia

**DOI:** 10.1136/bmjopen-2025-099536

**Published:** 2025-09-10

**Authors:** Molly Butler, Emma Veltman, Donna Lyon, Digsu Negese Koye, Phoebe Fitzpatrick, Eva Alisic, Luke Ney, Anna Goode, Genevieve Healy, Caitlin Hitchcock

**Affiliations:** 1 Melbourne School of Psychological Sciences, The University of Melbourne, Melbourne, Victoria, Australia; 2 Faculty of Fine Arts and Music, The University of Melbourne, Melbourne, Victoria, Australia; 3 The University of Melbourne School of Population and Global Health, Melbourne, Victoria, Australia; 4 Queensland University of Technology, Brisbane, Queensland, Australia; 5 The University of Queensland School of Human Movement and Nutrition Sciences, Saint Lucia, Queensland, Australia

**Keywords:** Gender-Based Violence, Peer Group, MENTAL HEALTH, Psychosocial Intervention, Exercise

## Abstract

**Introduction:**

Sexual and gender-based violence can have long-term impacts on the physical and mental health of survivors, with demonstrated impairments to immune, endocrine and nervous systems, and increased risk of chronic conditions such as cardiovascular disease, depression and post-traumatic stress. Moreover, survivors commonly experience low self-efficacy and lack of perceived control over their lives. Creating space for survivors to feel empowered through a multidimensional approach to health promotion, considering both physical and psychological influences on health, is necessary to reduce chronic disease.

**Methods and analysis:**

In this type II hybrid effectiveness-implementation cluster randomised controlled trial, we evaluate a novel peer-led intervention that combines expressive writing and trauma-informed boxing, Left Write Hook, against trauma-informed boxing alone—an intervention approach that is currently accessible in the community and has been shown to improve both mental and physical health. 20 clusters of 8–10 adults (n=150) with a self-reported history of child sexual abuse or other gender-based violence will be recruited in Melbourne, Australia, through health services and the community. Clusters will be randomly assigned to complete either 8× weekly group sessions of Left Write Hook (intervention) involving both expressive writing and trauma-informed boxing led by a trained peer facilitator, or 8× weekly group boxing sessions led by a trauma-informed boxing facilitator (control). Implementation will be evaluated against the PRISM Reach Effectiveness Adoption Implementation Maintenance (RE-AIM) framework. The primary effectiveness outcome is change in self-efficacy from preintervention to postintervention (8 weeks). Secondary effectiveness outcomes are changes in symptoms of complex post-traumatic stress disorder, trauma-related cognition and indicators of physical fitness (strength, flexibility, aerobic fitness and balance). Assessment will be completed online or over the phone with a member of the research team at preintervention (0 weeks), postintervention (8 weeks) and at 1 month following completion of the intervention (12 weeks). The primary implementation outcome is the fidelity of the train-the-champion implementation strategy for intervention training and delivery, and the secondary implementation outcome is adoption of the intervention and training delivery.

**Ethics and dissemination:**

Ethical approval was received from the Human Research Ethics Committee of The University of Melbourne (2024-28998-60131-11) and the Alfred Hospital Ethics Committee (110810). Results will be disseminated via publication in a peer-reviewed journal, and data will be made available via Open Science Framework at the conclusion of the trial.

**Trial registration number:**

ACTRN12624000862549.

**STRENGTHS AND LIMITATIONS OF THIS STUDY**

- Survivor-designed and survivor-led peer intervention; consumer contributions shape the study design (outcomes, measures).
- The hybrid effectiveness-implementation design allows for insights to inform broader scale-up of peer-led interventions.
- The study employs a range of multidimensional outcome measures, including self-report mental health measures, physical and biological measures and semistructured qualitative interviews.
- This study is limited by the inability to mask those completing the in-person physical health assessments.
- Recruitment is restricted to a small number of sites, limiting implementation implications.

## Background

 Child sexual abuse and other forms of gender-based violence constitute a major public health concern with well-documented short- and long-term effects on the physical and mental health of survivors.^1 2^ Gender-based violence includes intimate partner violence, sexual violence and other harmful acts directed at individuals on the basis of their gender.^3^ It disproportionately impacts women, with 35% of women experiencing sexual or domestic violence in their lifetimes,^3^ as well as gender-diverse individuals, who face equivalent or even greater risk.^4^ Gender-based violence, particularly during childhood, increases the likelihood of chronic mental (eg, depressive and post-traumatic stress) and physical (eg, cardiovascular and respiratory) health disorders.^5 6^ The link between gender-based violence and chronic health conditions in adulthood represents a complex interplay of physical and psychological factors. Survivors have higher levels of stress, which can increase engagement in health risk behaviours such as reduced physical activity, substance use and unhealthy diet,^7^ each of which uniquely contributes to the development of chronic disease.^8 9^ This is in addition to the demonstrated negative impact of abuse (notably sexual abuse) on functioning of the immune, endocrine and nervous systems.^10^,^12^ These issues are compounded by systemic biases wherein the impact of trauma on physical health often goes unrecognised.^13^ A multidimensional approach towards health promotion, which dually considers physical and psychological health, is sorely needed to reduce chronic disease in this population.^14^

### Existing interventions

There are a number of gold-standard treatments that have established benefits for survivors, such as trauma-focused cognitive behavioural therapy and eye movement desensitization and reprocessing (EMDR).^15^ However, there are considerable barriers to accessing such treatments, notably due to the limited number of professionals trained to deliver them.^16 17^ Even if these barriers are overcome, these interventions predominantly emphasise alleviation of psychological symptoms.^15^ However, survivors also experience alterations in physiological stress responses, such as heightened threat detection and modified stress hormone patterns.^18^ Some evidence suggests that cognitive-based therapies do not adequately address these somatic impacts of trauma, with symptoms such as dissociation, insomnia and pain difficult to shift.^19^,^21^ Consequently, survivors often report feeling underserved by traditional approaches to mental healthcare that fail to address individual needs and the lasting impacts of trauma, leading to substantial treatment dropout.^22^ ^23^ This highlights the need for alternative treatment approaches that overcome the limitations of existing clinician-delivered, cognitive-based therapies.

Here, we take a coproduction approach to evaluating a peer-delivered intervention for survivors of gender-based violence.^24^ In doing so, we seek to centre lived experience voices in setting research priorities and methodological design, ensuring that the peer-led nature of the intervention is also reflected in the research evaluating the intervention. By working closely with the survivor-led charity which designed and delivers our evaluated intervention, we provide a direct pathway for implementing our research outcomes for survivors of gender-based violence.

### Trauma-informed, peer-led interventions

Trauma-informed, peer-led interventions that centre survivors’ expertise in their own healing show potential to overcome the limitations of existing clinician-delivered therapies.^25^ Trauma-informed care encompasses principles of safety; trustworthiness and transparency; peer support; collaboration and mutuality; empowerment, voice and choice and cultural, historical and gender issues.^26^ Peer-led support programmes fit within this framework to create environments that privilege mutual support and relational healing while reducing power imbalances inherent in traditional therapeutic relationships.^25 27^ These survivor-centred approaches, facilitated by individuals with lived experience rather than mental health professionals, offer a promising alternative that can enhance treatment acceptability and help to overcome traditional barriers to care.^25^ Rather than positioning trained clinicians as agents of empowerment, peer support recognises survivors as central agents of their own recovery.^28^ Although growing evidence supports peer-led approaches for survivors, implementation challenges have hindered their adoption.^29^ Thus, scaling peer-led interventions requires systematic evaluation of both effectiveness and implementation outcomes. Essential implementation components include developing comprehensive training protocols and establishing accessible supervision structures that enable peer facilitators to deliver interventions safely (for both themselves and those they support) and effectively.^30 31^

### Writing-based interventions

There is a strong evidence base for the efficacy of writing-based interventions such as written exposure therapy,^32^,^34^ wherein survivors write expressively about the trauma with the guidance of a trained support person.^32^ As written-based therapeutic interventions require less specialist training and clinical expertise to deliver relative to current first-line treatments (eg, EMDR),^15^ they are well suited to peer delivery. Aligned with current first-line treatments, writing-based therapies facilitate the cognitive processing of trauma-related schema^35 36^ while developing capacity to tolerate and process difficult internal experiences.^34^ Writing about trauma in a supportive peer environment may strengthen survivors’ coping skills by teaching them that their trauma memories are not dangerous, that distress associated with recalling trauma is both tolerable and transient and that it is possible to develop new perspectives on trauma and its meaning.^32^ In addition to established effects for psychopathology, including reductions in symptoms of post-traumatic stress disorder (PTSD), anxiety and depression,^37^,^39^ there are some suggestions that expressive writing is associated with improvements for both objectively assessed and self-reported physical health outcomes.^40^ While writing offers valuable benefits for trauma processing,^35 36^ an important therapeutic goal for survivors is to reconnect with and regain agency over their bodies.^41^ Moreover, recent evidence has demonstrated that augmenting trauma-focused exposure therapy with aerobic exercise can improve therapeutic outcomes.^42^ Accordingly, incorporating embodied practices that help survivors ground themselves in the present and reconnect with their physical self after engaging with trauma memories through writing may therefore enhance therapeutic effects.

### Body-based interventions

Integrating body-based and cognitive techniques may better address survivors’ diverse needs and promote sustained recovery outcomes.^43 44^ There are a number of body-based therapeutic techniques which are beneficial for trauma survivors.^44^ In particular, non-contact boxing offers promise as a modality to help survivors reconnect with their bodies and ground into the present. Non-contact boxing combines high-intensity training and bodily awareness through techniques including stretching, skipping, shadowboxing, pad work and bag work.^45^ It is shown to foster an enhanced sense of empowerment and ability to locate healthy aggression in survivors,^46^ along with increases in agency, resilience, self-esteem and perceived physical ability.^47^ Encouragingly, exercise-based adjuncts fit easily into peer-delivery models and have demonstrated impacts for survivors in improving symptoms of PTSD, depression and anxiety.^45^ Improvements extend beyond mental health, with evidence of physical activity interventions reducing risk of chronic disease (including cardiovascular, respiratory and type 2 diabetes) by up to 80%.^48^ Given potential benefits for improving both mental and physical health in survivors, combining this physiological-based approach with an established psychological technique such as expressive writing may help to enhance outcomes for survivors.

In sum, current research suggests that a programme that incorporates both a physical health component and a psychological component may increase treatment benefits for trauma-exposed individuals.^42 49^ However, the unique combination of expressive writing and non-contact boxing is yet to be evaluated. These components can be delivered in a group-based setting, conferring additional benefits such as decreasing stigma and shame and enhancing social connection.^50^ Critically, this combined approach is suited to peer delivery, and taking a peer-led approach may further increase the acceptability and accessibility of support programmes.^51^

### Left Write Hook

Here, we seek to trial a novel intervention, Left Write Hook, which combines expressive writing and non-contact boxing within a peer-delivery model. Left Write Hook is a survivor-designed intervention, developed independently by a survivor of child sexual abuse in the community (ie, rather than formally developed within a research context). The programme was borne from the founder’s own lived experience of gender-based violence and reflection on an unmet need for connection with others who shared similar experiences to her own. In considering how to foster a space in which survivors could come together as part of a shared journey towards healing, she recognised the profound impact of both creative practices (eg, expressive writing, as opposed to written exposure techniques) and movement-based practices (eg, boxing) in her own healing journey and noted that such practices were often lacking from commonly offered psychological therapies (eg, cognitive behavioural therapy, cognitive processing therapy and EMDR). Initial efforts included establishing a support group that came together to practise writing and boxing in a peer-led manner and which received significant support within her local community. Subsequently, the Left Write Hook founder sought to formalise the programme through the development of a manualised programme, ultimately establishing a charity which is funded by philanthropic contributions to deliver the programme across Victoria. Concurrently, the Left Write Hook charity sought to partner with researchers to formally establish the feasibility and acceptability of the intervention.^52^

Left Write Hook is a survivor-led programme delivered via 8× weekly 2-hour group-based sessions in a community setting. Each session has two components: expressive writing and trauma-informed non-contact boxing. Participants are invited to write to prompts designed to encourage meaning-making from past experiences, with the option of sharing with the group to foster expression and validation. Trauma-informed non-contact boxing combines the approach of trauma-informed care with the physical activity of boxing, inviting participants to connect with their bodies through grounding, stance and focus on purposeful movement in a way that caters to their individual needs. Left Write Hook has the potential to expand beyond the established benefits of non-contact boxing alone through three key enhancements: the peer facilitator model encourages social connection and belonging; the additional writing component enables individuals to reclaim their life narratives and process difficult internal experiences; and the empowerment focus carries themes from the writing through to the boxing components to encourage grounding into the self and expression through physical movement.

As noted earlier, prior research on the Left Write Hook has been conducted concurrently with the dissemination of the programme in the local community within which it was established. After the programme was developed, a pilot study was conducted with adult survivors of child sexual abuse to investigate the efficacy of the programme and potential for future expansion.^52^ Large effect sizes were seen for preintervention to-postintervention improvement on a self-report measure of emotional, social and psychological well-being (Cohen’s *d*=0.99), alongside clinically meaningful reductions in PTSD symptomatology (Cohen’s *d*=1.02).^52^ Survivors who completed the programme experienced a large increase (Cohen’s *d*=1.48) in the self-reported ability to assert oneself, affect one’s environment and perform actions.^52^ This increased self-efficacy was reflected in qualitative analysis which demonstrated that the expressive writing promoted themes of participant empowerment, validation and connection to one’s body (eg, ‘I want to fix my body and my mind and feel strong’), with writing outputs reflecting that these themes evolved positively across the course of participation. Demonstrating feasibility and acceptability, validation of participants’ stories and feelings also emerged as a key qualitative theme, and 80% of participants completed all sessions and research measures. A formal evaluation of programme efficacy, acceptability and feasibility is now needed.

### Self-efficacy

There are multiple processes of change which may drive improvements in both mental and physical health, and our pilot data suggested that Left Write Hook may exert its effects by empowering survivors. Here, we evaluated programme effects on self-efficacy for a number of reasons. First, the General Self-Efficacy Scale^53^ has strong psychometric properties and incorporates elements of agency, assertiveness and control to assess individuals’ belief in their overall capability to succeed—factors which all improved in our pilot data. Self-efficacy is persistently low in survivors, reflecting pervasive perceptions of a lack of self-agency and power over their own lives.^54^ Similarly, survivors may mentally disconnect from their bodies (ie, dissociate) in order to cope with their experiences and can, as a result, feel a lack of agency over their own bodies.^55^ Critically, self-efficacy is longitudinally associated with mental and physical health outcomes in both community and trauma-exposed populations, with greater self-efficacy linked to decreases in PTSD symptoms, self-report pain, fatigue and substance use, alongside increases in physical activity.^56^,^58^ Finally, aligned with our coproduction approach, survivors were central to deciding the primary outcome for this trial. Our lived experience advisors stressed the importance of an outcome measure that is recovery-oriented rather than symptom-based, which reflects how the survivor is moving forward with their life rather than emphasising ongoing challenges. We therefore considered intervention efficacy on self-efficacy, with secondary outcomes of mental and physical health.

### Implementation

Beyond establishing treatment efficacy, considering implementation is essential for new interventions. A significant gap often develops when translating treatments developed within controlled research settings into real-world mental health settings.^59^ Treatment fidelity, the degree to which interventions are delivered as intended, presents a fundamental challenge, as real-world delivery often deviates substantially from original protocols.^60^ These challenges may be particularly pronounced in peer-delivery models, where facilitators have diverse professional backgrounds and experience.^61^ Additionally, working with survivors of gender-based violence who often have complex and diverse needs may create tension between protocol adherence and dynamic, person-centred care. Maintaining intervention fidelity is essential not only to ensure treatments achieve their intended effects but also to inform effective scaling to new contexts.^60^ Therefore, systematically assessing fidelity across both training and delivery phases is fundamental to ensuring new interventions can be implemented safely and effectively in diverse settings.^62^ Our use of a hybrid effectiveness-implementation trial design allows dual consideration of a primary effectiveness (individual-level) and primary implementation (individual level and site-level) outcomes,^62^ enabling formal evaluation of both the potential benefits of the intervention for survivors and the acceptability and feasibility of intervention delivery within established organisations (health services and charities) which support survivors.

### The current study

We aim to complete a hybrid effectiveness-implementation cluster-randomised control trial to both evaluate the impact of Left Write Hook on the self-efficacy of adult survivors of child sexual abuse and gender-based violence and concurrently develop and evaluate the fidelity of a train-the-champion implementation strategy for delivery in new settings. Alongside assessing effectiveness, an implementation evaluation is essential to establish to inform intervention scalability as well as address apprehensions around peer support programmes for survivors.^27^ We hope that insights into the implementation of Left Write Hook will help to inform training and delivery models of peer-led interventions for survivors more generally.

We compare Left Write Hook to a control group of trauma-informed non-contact boxing only, delivered by a personal trainer (rather than a fellow survivor), akin to the type of current evidence-based programme survivors can access in the community. The resource (ie, both interventions able to be delivered by non-psychologists) and time commitment for participants were matched between trial arms. We hypothesise that those completing Left Write Hook will experience a greater improvement in self-efficacy, relative to the control intervention, at postintervention.

## Methods

### Study design

This is a Type II hybrid effectiveness-implementation two-arm cluster-randomised controlled trial comparing Left Write Hook (intervention) to trauma-informed non-contact boxing-only (control). The primary effectiveness outcome measure will be self-efficacy at the primary endpoint of postintervention (8 weeks). Alongside the Left Write Hook programme, we aim to concurrently assess the implementation of ‘champion training’, a Trauma-Informed Writing and Boxing Facilitation programme for new Left Write Hook facilitators. The primary implementation outcome will be fidelity of intervention training and delivery of both the Left Write Hook and the boxing-only conditions within the trial, across different facilitators and sites. Fidelity refers to the degree to which an intervention or programme is delivered as intended.^60^ A hybrid trial design is appropriate for our research as it evaluates both effectiveness and implementation outcomes, accelerating the potential for broader implementation.^63^

### Study aims

In this study, we aim to evaluate the effectiveness of the Left Write Hook programme in improving self-efficacy (primary effectiveness outcome), and, subsequently, mental health and modifiable indicators for chronic disease (secondary effectiveness outcomes) in adult survivors of child sexual abuse and other gender-based violence. We dually aim to determine the fidelity of a train-the-champion implementation strategy for intervention training and delivery (primary implementation outcome).

### Study setting

Trial sites will be local community or health services. We anticipate 3–5 trial sites, each hosting a minimum of one intervention and one control group.

#### Site recruitment

Sites will be recruited from public mental health services and not-for-profit organisations which support trauma survivors in the state of Victoria, Australia. For inclusion, sites must consent to cluster randomisation of participants within their site, agree to advertise the trial to their service users and provide a site liaison who will work with the research team to manage intervention delivery and complete a semistructured interview to evaluate implementation outcomes at trial end. There is no minimum number of participants that each site must provide. Programme facilitators will travel between sites to deliver the programme. Equipment has been designed to be portable for ease of movement between sites.

#### Participant recruitment

Trial participants will be adults (n=150) recruited via participating sites (ie, government and not-for-profit health services with a dedicated trauma support service) alongside online advertisements. A documentary detailing the development of Left Write Hook will also raise public awareness of the intervention,^64^ and flyers advertising the trial are displayed at cinemas screening the film. Following input from lived experience advisors and in line with principles of trauma-informed care,^26^ we have deliberately kept our inclusion criteria broad to ensure individuals maintain agency over their participation. To be included in the trial, participants must be 18 years or older, female-identifying or gender diverse, self-report a history of child sexual abuse or gender-based violence, including family violence, and be willing to provide contact details for their GP (General Practitioner) to enable the research team to make the GP aware of participation and to coordinate support if needed during participation. For the purposes of this study, the term ‘gender-based violence’ is inclusive of any experience of sexual violence or any physical, sexual or emotional violence from an intimate partner or family member in either adulthood or childhood. Exclusion criteria are high levels of suicidality in need of more crisis management, evaluated by a clinical psychologist and informed by the Suicidal Ideation Attributes Scale.^65^ All participants complete an intake interview with a clinical psychologist trained in risk formulation to collaboratively decide whether to participate. During the interview, the clinical psychologist queries physical health, mental health and situational factors (eg, ongoing violence) that may impact an individual’s safe participation. Safe participation means that involvement in the programme does not negatively impact an individual’s physical or mental health, does not place them or others at risk of harm and does not interfere with ongoing evidence-based treatments (eg, participants undergoing EMDR may choose to delay participation until treatment is complete to avoid potential interference from the expressive writing component of Left Write Hook with memory reprocessing). Where safety concerns are identified, the clinical psychologist may recommend that the individual delay participation until their circumstances are more stable; for example, once an injury is healed or external supports are in place. We initially preregistered inclusion criteria to include a requirement for a letter of support from the GP; however, we decided to remove this to enhance equity of participation (ie, in situations where the GP has been non-responsive). Instead, the participant will provide contact information for their GP and any other mental health professionals, with consent sought for them to be contacted if safety concerns arise in the intake interview or during the programme. As per our risk management protocol (see online supplemental Materials for full protocol), if the trial team identifies safety concerns, the GP or other health professionals will be contacted to confirm that the participant can take part safely and to ensure continuity of care. Where the GP or other health professionals involved in the person’s care express concerns about safety of participation, this will be discussed with the individual so that they are aware of possible risks related to their involvement and they will be invited to participate once their professional supports advise it is safe to do so.

### Procedure

**Figure 1:**
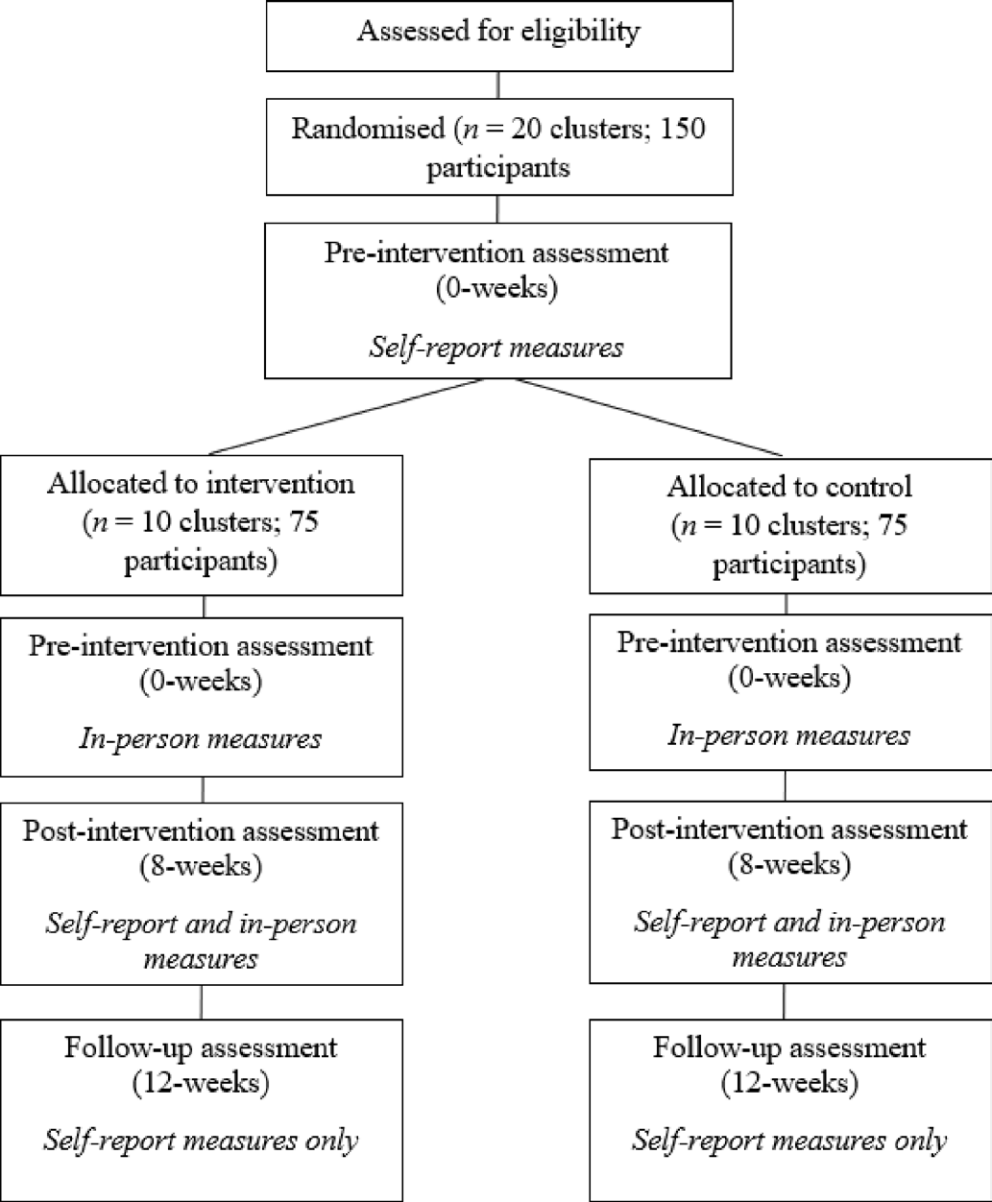
CONSORT diagram of participation. CONSORT, Consolidated Standards of Reporting Trials.

To determine eligibility, potential participants complete an intake interview with a clinical psychologist prior to enrolment in the trial. All enrolled participants will complete assessments at preintervention (0 weeks), postintervention (8 weeks) and at 1 month following the completion of the intervention (12 weeks). Self-report assessments can be completed at home or on the phone with a member of the research team. In-person assessments are taken by the programme facilitator immediately prior to the first session and immediately following the final session. Detailed information about the methodology and timing of assessments is outlined in figure 1. Eligible participants will not have to pay for the Left Write Hook or boxing-only programmes. Left Write Hook is currently accessible in the community (delivered via a survivor-led charity) through a suggested ‘pay what you feel’ donation of up to AUD$450 to the Left Write Hook charity. Participants also receive a AUD$20 Giftpay voucher for each of the three online assessments they complete. Those in the boxing-only condition will be offered participation in the Left Write Hook programme at the end of the trial.

### Randomisation and blinding

Cluster randomisation will use a block procedure for groups of 8–10 participants using computer-generated quasi-random numbers. Randomisation will be stratified by site, for between-site equity in the number of groups in each arm. The study will employ a single-masked design. This will be achieved by the allocation list being generated by a statistician independent from the research team. Study biostatisticians will remain masked until the database has been cleaned, a masked data review has taken place, and the database is ready for analysis.

### Intervention

#### Left Write Hook

Left Write Hook is delivered via 8× weekly 2-hour group-based sessions in a local gym/sports centre or community room. Sessions are led by two paid peer facilitators: a lead facilitator, assisted by an assistant facilitator, both of whom are survivors themselves. All peer facilitators are paid in line with industry rates for casually employed facilitators of exercise-based interventions. Each session has two components: expressive writing and trauma-informed non-contact boxing. An outline of the full programme, including session themes, is shown in table 1.

**Table 1:** Outline of Left Write Hook programme

Week	Theme	Primary session goal
1	Grounding	Help people feel comfortable in the space and instil participants with a sense of purpose and feeling of being grounded.
2	Balance	Teach participants how to channel their emotions through the boxing. Create a safe environment for difficult emotions.
3	Expression (fluidity)	Help guide participants through difficult emotions and help them feel comfortable expressing themselves through writing and boxing. Give participants a healthy outlet.
4	Lightness	To have participants feeling lighter and more accomplished. To have participants feeling confident with their boxing.
5	Taking back your power	To have participants feeling confident in their boxing capabilities. To give participants the ability to harness their power.
6	Fighting Back	Give participants the building blocks to defend themselves. Have participants feel confident with weaving and pad work. Have the participants understand and express healthy aggression.
7	Rewriting narratives	To have participants take back their personal power
8	Imagining futures	To have participants be ready for the next steps in life once the programme ends, whether their next step is to return, become a facilitator or leave with a more positive outlook on life moving forward.

Writing prompts reflect the theme of each session.

The first hour involves participants writing to recovery-focussed prompts designed to reclaim their life narrative and encourage meaning-making (eg, ‘fighting back means…’), with the option of sharing with the group to encourage expression and validation. Writing prompts reflect the theme of each session. Participants are invited to write about anything that comes to mind for them in response to the writing prompts and session theme. While this means that they are not required to write about past trauma and may choose to write about any topic, participants do predominately choose to write about their experiences of trauma or its impact on them. Peer facilitators guide the expressive writing and also participate in the writing and sharing. Next, participants complete a 45-min trauma-informed non-contact group boxing session. Peer facilitators guide participants to connect with their body and locate an empowered state, carrying through the theme from the expressive writing to guide participants’ focus on stance, punch, direction and movement. Each session ends with 15 min of grounding and reflections. Details on how the principles of trauma-informed care are embedded within the programme are outlined in table 2.

**Table 2:** Examples of trauma-informed considerations across interventions and research procedures

Programme element	Trauma-informed principle(s)^25^
*Intake*	
Use inclusive and easy to understand language	Empowerment, voice and choice
Provide clear and detailed information on what the programmes involve	Safety, trustworthiness and transparency
Collaborative decision-making with potential participants regarding programme suitability at this time	Empowerment, voice and choice
Establish confidentiality and safety regulations	Safety, trustworthiness and transparency
Let survivors choose to share or withhold	Empowerment, voice and choice
*Both Left Write Hook and boxing-only interventions*	
Female or gender-diverse facilitators	Empowerment, voice and choice
All facilitators trained in trauma-informed facilitation	Safety, trustworthiness and transparency
Venue selection (accessibility, private space, limiting mirrors, quiet break-away space)	Safety
Invitational facilitation style to promote agency and choice	Safety, empowerment, voice and choice
Facilitators complete in-person research assessments, not trial staff	Safety, trustworthiness and transparency
Sessions begin with an acknowledgement of country, which acknowledges the ongoing impact of generational and cultural trauma in colonised Australia	Cultural, historical and gender issues
Acknowledgement that boxing might activate discomfort	Safety, trustworthiness and transparency
Clear description of session structure at the beginning of each session	Safety, trustworthiness and transparency
Non-contact and non-violent approach to boxing	Safety
Modifications available and participants encouraged to go at their own pace	Empowerment, voice and choice; collaboration and mutuality
Focus on progress not performance	Empowerment, voice and choice
Build trust through consistency and established routines	Trustworthiness and transparency
*Left Write Hook only*	
Peer-led	Collaboration and mutuality; peer support
Cocreation of group values in week 1	Collaboration and mutuality
Facilitators write to prompts and share alongside participants	Collaboration and mutuality; peer support
Choice in writing prompts and activities	Empowerment, voice and choice
Invitation to locate healthy aggression	Empowerment, voice and choice
Non-censored space; group makes collaborative decision around what details are shared, and this is revisited throughout the programme	Empowerment, voice and choice; collaboration and mutuality

Trauma-informed principles as per Trauma-Informed Care in Behavioral Health Services, Substance Abuse and Mental Health Services Administration, 2014.

#### Active control: boxing-only

Participants randomised to control will complete 8× weekly group sessions of trauma-informed non-contact boxing, without writing and without a specific focus on empowerment, to control for the positive effects of exercise on self-efficacy. Each week, participants will complete a 45-min group boxing session, led by a boxing instructor (ie, intervention is not peer-led) at the same sites as the intervention arm. This replicates the type of trauma-informed exercise class that survivors could access in their local community. Boxing was selected as the exercise modality based on consumer input that identified it as the most acceptable exercise option for an active control, particularly in the context of consenting to randomisation. The control group therefore represents trauma-informed exercise options as usual, without three unique components of the Left Write Hook intervention: the peer-led nature of intervention, expressive writing and the focus on empowerment that links from the expressive writing to the trauma-informed non-contact boxing component to encourage expression through physical movement. Trauma-informed considerations for the boxing-only group are outlined in table 2.

#### Facilitator and participant support

Facilitators of both the Left Write Hook and boxing-only programmes are supported by fortnightly group supervision sessions with a clinical psychologist. The clinical psychologist is on call for facilitator and participant support during and after all sessions for both the intervention and control groups. They also offer debriefs to both facilitators and participants via phone call or Zoom on an ‘as needed’ basis. Left Write Hook facilitators additionally have access to a community of practice, facilitated through WhatsApp by the Left Write Hook charity. This community of practice is not monitored by the research team.

#### Champion training

**Figure 2:**
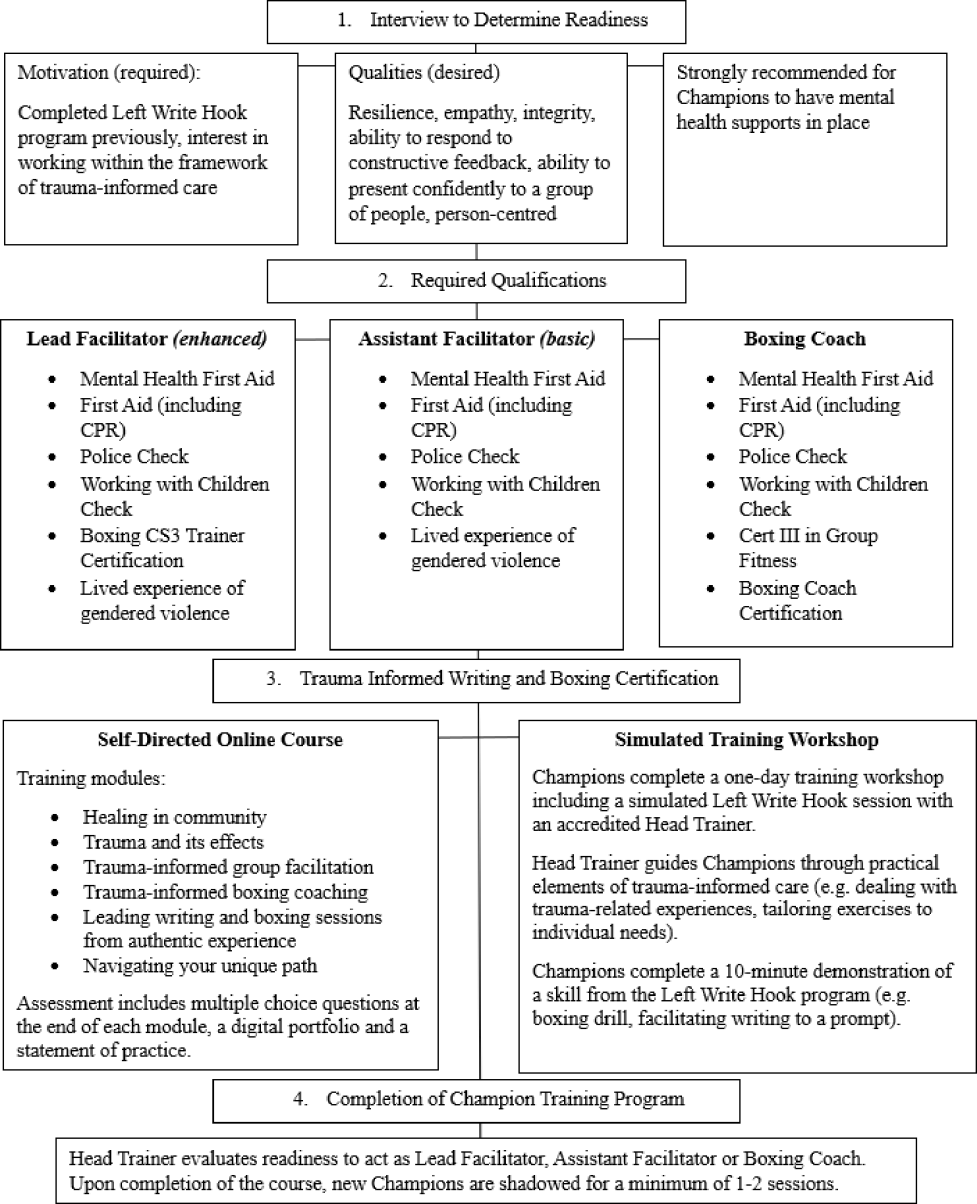
Flowchart of Champion Training Journey. CPR, Cardiopulmonary resuscitation.

Alongside the Left Write Hook intervention, we are assessing the implementation of a champion training programme (see figure 2 for overview of training components) for new Left Write Hook facilitators. Champions may train to perform one of three roles: lead facilitator, who holds enhanced qualifications; assistant facilitator, who primarily provides peer support and only holds basic qualifications or boxing coach, who has a boxing certificate and a group fitness bridging certificate (which are both completed externally), plus completed the trauma-informed boxing component of the Left Write Hook training programme. A lead or assistant facilitator can also act as a boxing coach (ie, deliver the boxing component of the intervention), if they have completed the appropriate certification. Certification is verified by Left Write Hook’s head of operations. Past Left Write Hook participants who have met these requirements are invited to enrol in a 6-week, self-directed online training course in Trauma-Informed Writing and Boxing Facilitation. The training course involves six online modules that guide prospective champions through the essential components of facilitating a Left Write Hook programme (module content outlined in figure 2). Modules are comprised of a mix of multimedia resources, educational videos, group discussion boards and assessments (multiple-choice questions, a digital portfolio, a statement of practice). At the completion of the training programme, collaborative discussion between Left Write Hook’s head of operations and champions evaluates readiness to act as a lead facilitator or whether beginning as an assistant facilitator is preferable. Champions become formal peer facilitators once programme requirements are complete (figure 2 placed here).

### Positionality statement

 Our core research team is made up of predominately white, cisgender women, including clinical psychologists, researchers and experts by lived experience. Some team members draw on both lived and learnt expertise, though their primary roles in this study are grounded in academic and clinical perspectives. We acknowledge that these shared and divergent positionalities shape our approach to research, including study design, data collection, participant engagement and interpretation of findings.

### Patient and public involvement statement

With a focus on the coproduction of research, this trial has been designed to actively involve and empower adult survivors of gender-based violence. The intervention has been designed and is delivered by survivors. Moreover, the trial has been designed to build the capacity of consumers to take ownership of the Left Write Hook intervention and to use their experiences to support others. In addition to the Lived Experience co-lead, the research has been codesigned by a paid Lived Experience Advisory Group (LEAG), comprising eight individuals who have experienced gender-based violence, including both those who have and have not participated in the Left Write Hook programme. The LEAG convenes each quarter to provide their perspectives and guidance. Their contributions shape the study design (outcomes, measures), inform the structure of the champion training and coproduce questions for semistructured interviews with intervention participants, facilitators and site partners. A member of the LEAG also sits on the Trial Steering Committee. This partnership supports critical engagement with dimensions of experience not wholly reflected in the core research team, particularly related to power, race and representation.

### Outcome measurement by RE-AIM dimensions

Data will be collected from the site liaison, programme facilitators and participants, with data collected via surveys, interviews, in-person measures, site visits and website analytics. Evaluation has been mapped to the PRISM RE-AIM framework^62 66^ to understand the context for implementation and indicators of Reach (individual participation in intervention), Effectiveness (intervention’s impact), Adoption (participation of sites/staff in intervention), Implementation (degree to which intervention is delivered as intended) and Maintenance (long-term intervention effects and degree to which intervention becomes institutionalised) in community settings. Comprehensive information on outcome measurement and data collection time points is presented in the online supplemental materials (Table S1).

#### Context

Information about the implementation and sustainability infrastructure (eg, resource availability, funding) at each site will be collected via site visits and interviews with the site liaison, with data collected preimplementation and at the end of the trial period. Perceived barriers and facilitators to implementation will be assessed by the pragmatic assessment context tool^67^ completed by the site liaison at preimplementation.

#### Reach

Reach will be assessed at the individual level and reported in terms of the number of people who expressed interest in taking part in a programme and the number and demographic characteristics of the people that were assigned to a programme (ie, treatment or control), completed the assigned programme and withdrew from the assigned programme, as well as the programme facilitators. Data will be collected from the intake interview and the preintervention survey. Demographic characteristics collected will include: age, gender, caring responsibilities, language spoken at home, Aboriginal and Torres Strait Islander status, NDIS (National Disability Insurance Scheme) status, work status, health conditions, access to treatment and services. Participants and facilitators will be asked to report any changes in the modifiable factors at the 8-week and 12-week assessments.

#### Effectiveness

Evaluation of effectiveness will be conducted at the individual level (participant and facilitator, including withdrawals) to determine the impact of the programme on psychosocial and physical health outcomes. Participants who withdraw from the study will still be invited to complete outcome measures. All outcome measures are outlined in the online supplemental materials (Table S1). The primary effectiveness outcome is self-efficacy, measured by participant score on the General Self-Efficacy Scale.^53^ Secondary effectiveness outcomes will include complex PTSD symptoms, trauma-related cognition (trauma memory quality and post-traumatic cognitions) and indices of physical health (strength, flexibility, aerobic fitness and balance). Exploratory effectiveness outcomes include depression and anxiety, social connectedness, mental well-being, quality of life, health behaviours (exercise habits, smoking and vaping frequency, alcohol intake and use of health resources) and physical health (blood pressure, chronic pain, sleep quality and stress hormones). Self-report measures will be administered online via Qualtrics, either at home in a comfortable environment, via phone call with a member of the research team or at the gym/sports centre with the support of a facilitator. Due to logistical constraints, preintervention in-person assessments (physical fitness, blood pressure and hair sample) will be completed immediately prior to the commencement of the first Left Write Hook or boxing sessions at the sport centre by an unmasked programme facilitator, with responses recorded via Qualtrics. Hair samples indexing stress hormones (see online supplemental Table S1 for details) will be collected by the programme facilitator, trained in collection procedure by a member of the research team. Samples will be wrapped in aluminium foil and stored in a plastic ziplock bag and kept at room temperature in a locked cabinet until analysis, as per recommendations in the field.^68^

#### Adoption

Adoption will be measured on the organisational level in terms of the per cent of sites approached that participate and their characteristics, the number and characteristics of the champions and the withdrawals (site/champion) from the trial and reasons for withdrawal. Characteristics of sites collected will include: organisation, postcode, number of members, staffing and existing programmes offered. At the individual level, adoption will be measured in terms of demographic characteristics of champions and will be collected as part of their pretraining survey. Characteristics captured will include age, gender, caring responsibilities, language spoken at home, Aboriginal and Torres Strait Islander status, NDIS status, work status, health conditions, access to treatment and services and experience/expertise. Data on withdrawals will be tracked by the project team.

#### Implementation

The primary implementation outcome is fidelity of the intervention and training delivery. Fidelity of the intervention across different facilitators and sites will be indexed by fidelity checklists. The lead facilitator and assistant facilitator will both complete fidelity checklists online via Qualtrics at the end of each session, which require them to endorse that each core component of the session has been completed. Checklists will index whether each activity was fully completed, partially completed or not done; whether there were additions or changes to the session and any challenges or issues affecting implementation. They will also request a rating of how confident the facilitator felt for the session, how engaged participants were in the session, whether facilitators approached the session in a manner consistent with the ethos of the programme (eg, holding a space for sharing without resolving or fixing, as well as supporting participants to modify the session to suit their individual needs and to bear witness to stories shared by their peers). Two trial participants will complete a similar checklist which asks whether each activity was fully or partially completed and requests a rating of whether facilitators approached the session in a manner consistent with the ethos of the programme. Accordance between facilitator and participant ratings will be assessed. Our primary fidelity outcome will be the mean proportion of intervention components marked as fully completed, averaged across facilitators, per intervention group.

Fidelity of the training delivery will be indexed by programme assessments which capture comprehension of module content, including a digital portfolio, a statement of practice as a facilitator/boxing coach and a simulated training workshop to demonstrate practical skills.

Semistructured interviews with sites, champions, peer facilitators and participants will be conducted by the research team using questions codesigned by the LEAG. Questions will be designed to explore perceptions among stakeholders, including that the programme and training are agreeable (ie,acceptability), as well as perceived fit and relevance of the programme in the local context (appropriateness). Costs associated with training and programme delivery will be tracked by the research team (sustainability). The barriers and enablers of implementation according to the Consolidated Framework of Implementation Research^69^ will be explored through interviews with sites, champions and participants. Adaptations to programme delivery will be captured through interviews with the site liaison and tracked by the project team. Semistructured interviews with participants and champions will be conducted by the research team in person or via Zoom and will be audio-recorded.

#### Maintenance

Maintenance of programme delivery will be assessed at the organisational level (sites) via interviews with the site liaison, with the primary indicator commitment to ongoing programme delivery at the end of the research funding period. At the individual level (participants), short-term maintenance will be assessed in terms of the effectiveness outcomes at 12 weeks, with maintenance intentions (ie, whether they intend to continue boxing and/or journalling) also captured at postintervention and 12 weeks via surveys.

### Sample size

A sample size of 128 participants (64 participants per arm, clustered into eight groups per intervention arm) would be required to have 80% power to demonstrate superiority of the Left Write Hook over the boxing-only intervention in terms of the primary effectiveness outcome of self-efficacy with a two-sided 5% significance level. This sample size is based on the following assumptions: clinically meaningful absolute treatment difference in mean change from preintervention to postintervention in self-efficacy score of 3 in favour of Left Write Hook (increase), a SD of 5.6 equal in each arm and at each time point (preintervention and postintervention)^70^ and an intraclass correlation of 0.02 to allow for possible clustering effects. Assuming a 15% dropout, a sample size of 150 participants is required (ie, 75 per arm clustered into 10 groups per intervention arm). This sample size represents 0.03% of the estimated number of gender-based violence survivors in the Melbourne population, based on Australian prevalence rates.^71^

### Statistical analysis plan

Results will be analysed and reported as per the Consolidated Standards of Reporting Trials statement for cluster randomised trials.^72^ A detailed statistical analysis plan will be developed by study statistician/s prior to database lock. Analysis will follow on an intention-to-treat basis according to the cluster’s randomisation allocation. No interim analyses are planned. Cluster characteristics and participant characteristics at preintervention will be summarised using descriptive statistics.

The primary effectiveness estimand for Left Write Hook is defined according to the addendum to the International Council for Harmonisation (E9) Addendum on estimands in clinical trials.^73^ Left Write Hook aims to answer the specific research question: does implementation of the Left Write Hook programme, compared with boxing only, over 8 weeks, improve self-efficacy in survivors, as measured by the change in the General Self-Efficacy Scale,^53^ including the effect of the postrandomisation (intercurrent events) outlined below. Intercurrent events are events that occur postrandomisation and may preclude the observation of the outcome variable or affect its measurement.^74^ Intercurrent events will be tracked by the study team during the study. Possible intercurrent events include the following: participants not adhering to intervention protocol (not attending sessions altogether), participants withdrawing before the end of the intervention, change in or concurrent therapies and/or medication and hospitalisation. Primary estimand attributes are outlined in the online supplemental materials (Table S2).

We will use linear mixed effects models to examine the change in the primary outcome of self-efficacy. The response variable will consist of preintervention and follow-up measures, and the model will include fixed effects for treatment group (intervention vs control) and the randomisation stratification factor (site). Random effects will be included to account for clustering at the participant and cluster levels. The absolute treatment difference in mean change from preintervention (week 0; baseline) to 8 weeks postrandomisation (ie, post-intervention) will be estimated with corresponding two-sided 95% CI and p value to evaluate the primary outcome. An adjusted treatment effect based on a model including potential confounding variables will also be obtained. Continuous secondary and exploratory outcomes will be analysed similarly to the primary outcome and targeting a similar primary estimand. Binary secondary and exploratory outcomes will be analysed using logistic regression with adjustment for site. Hair samples will be subject to mass spectrometry, with preintervention and postintervention differences analysed using paired sample t-tests. Quantitative data for the champion experience will be analysed descriptively only.

To determine implementation outcomes, qualitative data from semistructured interviews with participants, champions and peer facilitators will be analysed using NVivo, in line with reflexive thematic analysis.^75^ Themes will be interpreted in collaboration with the LEAG. Finally, the economic costs and benefits of Left Write Hook will be explored using Assessment of Quality of Life^76^ outcomes and costs of champion training and intervention delivery.

### Trial status and registration

Recruitment for the trial is currently open, having commenced in September 2024, and is expected to continue until June 2026. This trial was prospectively registered with the Australia New Zealand Clinical Trials Registry (ACTRN12624000862549) prior to participant enrolment.

### Ethics and dissemination

This trial is funded by an Australian National Health and Medical Research Council, Medical Research Future Fund Consumer-Led Award (2031217). The National Health and Medical Research professional and ethical guidelines will be adhered to throughout the study. Ethics approval has been obtained from the University of Melbourne Research Ethics Committee (2024-28998-60131-11) and Alfred Hospital Ethics Committee (ID 110810). The trial is sponsored by the University of Melbourne (2024-29092-54697-2). All participants will provide written informed consent prior to participating in the study. Any deviation from the protocol will receive approval from the ethics committee and will be reported in the published trial manuscript. No restrictions have been placed on the publication of results.

#### Monitoring and data management

Participant safety has been prioritised through the development of this survivor-designed and survivor-led intervention. The Co-Investigator team includes clinical psychologists who are experienced in this subject area and will manage any risk in accordance with the Clinical Risk Management Protocol (provided in online supplemental material). Any adverse events will be overseen by the Trial Steering Committee (TSC; chaired by an independent clinical psychologist not involved in the trial (Professor Hiller), with members of the LEAG), who will determine the suitability of continuing the trial (ie, determine stopping), along with reporting to the ethics committee and trials registry. The Trial Management Committee (TMC; trial lead, lived experience researcher and trial coordinator) will manage any arising risks for participants or trial completion, ensure timelines are met, manage data security and ethics reporting. The TMC will manage data, with oversight by the TSC. All daily trial management will be completed by the trial coordinator. Data checks will be completed by the trial coordinator to ensure data quality.

### Confidentiality

Data will be stored on encrypted password-protected servers and only accessible to the research team. Confidentiality will only be broken if required by law, if we are concerned for the safety of a participant or that of someone else. If this happens, the participant will be first notified. Names will be replaced in qualitative data, during transcription, to ensure anonymity. Identifiable data will be kept securely for 5 years and then destroyed, as per legal regulation. Participants can request that their data be deleted within this time frame. Deidentified data will be kept in perpetuity. Participants will provide unspecified consent for deidentified data to be shared via the Open Science Framework at the conclusion of the trial.

#### Safety aspects

No adverse events are anticipated as a result of the study. However, as all participants have experienced gender-based violence, there is a risk of suicidality and/or self-harm. This will be monitored using clinical judgement and structured risk assessment. The trial lead and trial coordinator are registered clinical psychologists experienced in the treatment and management of trauma-related mental health challenges and will supervise all staff to execute safety protocols if adverse events do occur. All participants must provide GP contact information prior to enrolment, and we will directly contact the GP if deemed necessary by the supervising psychologist to ensure safety. Intervention facilitators have access to fortnightly group-based supervision with a clinical psychologist and peer support through the community of practice. Risks will be included in annual progress reports submitted to the funder and ethics committees. Trial audits will be completed annually by the Trial Sponsor (University of Melbourne Clinical Trial Governance).

## Supplementary material

10.1136/bmjopen-2025-099536 online supplemental file 1
