# Supplementary material for: Left Write Hook: trial protocol for a community-based type II hybrid effectiveness-implementation cluster randomised controlled trial of a boxing and writing-based intervention for adult survivors of child sexual abuse and gender-based violence in Australia
Source: BMJ Open. 2025 Sep 10;15(9):e099536. doi: 10.1136/bmjopen-2025-099536 (PMC12519368; doi:10.1136/bmjopen-2025-099536)
Supplement: online supplemental file 1 [file bmjopen-15-9-s001.docx]

**Left, Write, Hook: Trial Protocol for a Type II Hybrid Effectiveness-Implementation Randomised Control Trial of a Boxing and Writing-Based Intervention for Adult Survivors of Child Sexual Abuse and Other Gendered Violence**

**Supplementary Materials**

**25 July 2025**

**Contents:**

Table S1

Table S2

Risk Protocol

**Table S1**

*Outcome Measurement by RE-AIM Dimensions*

| Dimension | Outcome | Measure | Method of collection | Time-point | Respondent(s) |
| --- | --- | --- | --- | --- | --- |
| Reach | Demographic characteristics | - | Intake interview, self-report survey | Pre-intervention (0-weeks)  Post intervention (8-weeks)  Follow up (12-weeks) | Participant  Facilitator |
|  | Referral pathway | - | Expression of interest self-report survey | Pre-intervention | Participant |
| Effectiveness |  |  |  |  |  |
| Primary Effectiveness Outcome | Self-efficacy | General Self-Efficacy Scale [1] | Self-report survey | Pre-intervention  Post intervention  Follow up | Participant  Facilitator (pre- and post- only) |
| Secondary Effectiveness Outcomes | Complex Post-Traumatic Stress Disorder | International Trauma Questionnaire [2] | Self-report survey | Pre-intervention  Post intervention  Follow up | Participant  Facilitator (pre- and post- only) |
|  | Trauma memory quality | Trauma Memory Quality Questionnaire [3] | Self-report survey | Pre-intervention  Post intervention  Follow up | Participant  Facilitator (pre- and post- only) |
|  | Trauma related cognition | The Post-Traumatic Cognitions Inventory [4] | Self-report survey | Pre-intervention  Post intervention  Follow up | Participant  Facilitator (pre- and post- only) |
|  | Physical fitness |  |  |  |  |
|  | Hand grip strength | Digital hand dynamometer | In-person | Pre-intervention  Post intervention | Participant |
|  | Flexibility | 30 second sit-to-stand test | In-person | Pre-intervention  Post intervention | Participant |
|  | Aerobic fitness | 3-minute step test | In-person | Pre-intervention  Post intervention | Participant |
|  | Static balance | 4-step balance test | In-person | Pre-intervention  Post intervention | Participant |
| Exploratory effectiveness outcomes | Depression and anxiety | Depression Anxiety Stress Scale-21 [5] | Self-report survey | Pre-intervention  Post intervention  Follow up | Participant  Facilitator (pre- and post- only) |
|  | Loneliness | UCLA 3-item Loneliness Scale [6] | Self-report survey | Pre-intervention  Post intervention  Follow up | Participant  Facilitator (pre- and post- only) |
|  | Social connectedness | Social Connectedness Scale-Revised [7] | Self-report survey | Pre-intervention  Post intervention  Follow up | Participant  Facilitator (pre- and post- only) |
|  | Wellbeing | Short Warwick-Edinburgh Mental Wellbeing Scale [8] | Self-report survey | Pre-intervention  Post intervention  Follow up | Participant  Facilitator (pre- and post- only) |
|  | Quality of life | Assessment of Quality of Life-8 [9] | Self-report survey | Pre-intervention  Post intervention  Follow up | Participant  Facilitator (pre- and post- only) |
|  | Health behaviours |  |  |  |  |
|  | 24 movement behaviour* | Time spent in five behaviours (sleep, sedentary time, moderate-to-vigorous intensity activity, other movement, standing: must sum to 24 hours) | Self-report survey | Pre-intervention  Post intervention  Follow up | Participant  Facilitator (pre- and post- only) |
|  | Smoking behaviour | Frequency of smoking and/or vaping in past 7 days | Self-report survey | Pre-intervention  Post intervention  Follow up | Participant  Facilitator (pre- and post- only) |
|  | Alcohol intake | Alcohol Use Disorders Identification Test [10] | Self-report survey | Pre-intervention  Post intervention  Follow up | Participant  Facilitator (pre- and post- only) |
|  | Access to health services | Frequency of visits to general practitioner, mental health clinicians, and attendance at hospital emergency in past four weeks. | Self-report survey | Pre-intervention  Post intervention  Follow up | Participant  Facilitator (pre- and post- only) |
|  | Physical health |  |  |  |  |
|  | Resting blood pressure | Automated blood-pressure cuff | In-person | Pre-intervention  Post intervention | Participant |
|  | Sleep quality | Insomnia Severity Index [11] | Self-report survey | Pre-intervention  Post intervention  Follow up | Participant  Facilitator (pre- and post- only) |
|  | Chronic pain | Brief Pain Inventory short form [12] | Self-report survey | Pre-intervention  Post intervention  Follow up | Participant  Facilitator (pre- and post- only) |
|  | Stress hormones (Arachidonoyl, ethanolamide, 2-arachidonoyl glycerol, oleoylethanolamide, palmitoylethanolamide, stearoylethanolamide, lineoylethanolamide, arachidonic acid, cortisol, cortisone  dehydropiandrosterone, dehydropiandrosterone-sulfate, progesterone, testosterone) | Hair sample | In-person | Pre-intervention  Post intervention | Participant |
| Adoption | Site participation and characteristics | - | Semi-structured interview (in-person or online) | Ongoing | Site liaison |
|  | Champion demographic characteristics | - | Self-report survey | Ongoing | Champions |
| Implementation |  |  |  |  |  |
| Primary implementation outcome | Fidelity of interventions | Fidelity checklists | Self-report survey | Ongoing | Participant (2 per session)  Facilitator |
|  | Fidelity of Train-the-Champion program | - | Program assessments, website analytics | Ongoing | Champions |
| Exploratory implementation outcomes | Acceptability | Perceptions among stakeholders that the program and training are agreeable | Semi-structured interview (in-person or online) | Ongoing | Participants  Facilitators  Site liaisons  Champions |
|  | Appropriateness | Perceived fit and relevance of the program in the local context | Semi-structured interview (in-person or online) | Ongoing | Participants  Facilitators  Site liaisons  Champions |
|  | Sustainability | Costs associated with training and program delivery | Direct observation | Ongoing | Research team |
| Maintenance | Maintenance of program delivery | Semi-structured interview | Semi-structured interview (in-person or online) | Ongoing | Site liaison |
|  | Maintenance intentions for intervention | Intention to continue boxing and/or journalling | Self-report survey | Post intervention (8-weeks)  Follow up (12-weeks) | Participants |

*Note.* *Criterion validity against activPAL (*r* = .35 sedentary, *r* = .40 standing, *r* = .58 moderate to vigorous intensity movement)

**Table S2**

*Primary Estimand Attributes*

| Attribute | Description |
| --- | --- |
| Treatment | Left Write Hook program (expressive writing and trauma-informed boxing) to boxing only (control), allocation by cluster randomisation. |
| Population | Female identifying or gender diverse survivors of childhood sexual abuse or other gendered violence aged 18 years or older. |
| Variable | Self-efficacy scores at 8-weeks post randomisation (i.e., at post-intervention) as measured using the General Self-Efficacy Scale. |
| Intercurrent events | Possible intercurrent events will be handled using the treatment policy strategy. Where possible, outcome data will be collected after intercurrent events occur. |
| Population level summary measure | The absolute mean difference in self-efficacy scores after 8-weeks of assignment to Left Write Hook program, relative to boxing only intervention. |

**Supplementary Materials: Risk Protocol**

# Left Write Hook Randomised Control Trial Risk Protocol

## Prior to study participation

Background information regarding the participant’s mental health and supports will be collected by a Clinical Psychologist during the intake interview, prior to their enrolment in the research. The intake interview will assess their suitability to participate, and to ensure that appropriate supports are in place throughout their participation in the study. Information collected will include:

- Current mental state
- Mental health history
- History of irritability, aggression or violence
- Substance use
- Suicidality as assessed via the Suicidal Ideation Attributes Scale
- Existing coping strategies and self-care plan

In addition to the above, all participants will be required to have existing mental health supports (e.g. a GP and mental health professionals if required) in place. Participant consent will be obtained for research staff to contact their GP and any mental health professionals involved their care to make them aware of participation in the program. Professional supports are asked to contact the research team if they have concerns about the individual’s involvement in the trial. Participants who do not have existing mental health supports in place, or whose support providers express concerns regarding their safety to participate, will not be enrolled to participate. Participants will also be informed that research staff will contact the GP and/or mental health professional(s) during study participation if the participant displays increased risk to self or others.

Similarly, participants who are assessed (as evaluated by a Clinical Psychologist and informed by the Suicidal Ideation Attributes Scale) as having a high level of suicidality in need of consistent crisis management will not be eligible to participate in the study. This is for their own safety. Rather, these individuals will be supported to connect with a mental health service. Again, research staff will contact the participant’s GP if participant consent is provided.

Any safety concerns identified by a Clinical Psychologist that may impact the safety of the individual or other group members during participation (e.g. ongoing experiences of trauma that could pose a risk within a group setting) will also be used to determine participant inclusion.

In all of the above cases, participant enrolments will be delayed until it has been established that they can participate safely.

Clinical notes regarding the above will be stored in password protected files on an encrypted server. Only the Trial Management Committee will have access to these files.

Prior to the first session, facilitators will be provided with a list of participants, along with a summary of relevant participant characteristics that may be helpful for supporting the participants, and ensuring that they can participate safely.

This will include;

- any physical health conditions (e.g., heart disease)
- mobility issues (e.g., balance)
- pre-existing injuries (e.g., recurrent join pain)
- any substance or medication use that may influence participation (e.g., insulin, Ventolin)
- prior experience with boxing
- participant identified triggers or trauma features that may influence engagement or be anticipated to evoke strong reactions during peer interactions (e.g., involvement of organised religion in abuse)
- high dissociative experiences
- active suicidal ideation
- any other details that the participant would like the facilitator to understand about them.

Both participants and facilitators will be informed that facilitators can contact the Clinical Psychologist with concerns for the participant’s wellbeing. Participant summaries will be shared verbally, by the Clinical Psychologist, to the facilitators to allow them to ask questions and explore management strategies that may be of use. The summaries will also be saved on the encrypted server, with facilitators only able to access the information for the participants in groups that they are facilitating.

## During participation in the study

Should participants experience distress during their participation in the study, the following steps will be followed.

| **Risk situation** | **Plan** |
| --- | --- |
| Clinical psychologist identifies concerns about the physical or mental health and safety of an individual during the intake call or during program participation. | Clinical psychologist to contact the individual’s professional supports and would require input from them before proceeding with enrolment. Where professional supports express concerns, or judgement of Clinical Psychologists on the research team deems participation unsafe, this will be discussed with the participant so that they are aware of possible risks related to their involvement. The person would then be invited to participate once their supports and/or the research team advise it is safe to do so. |
| Facilitator/coach has concerns about the safety and/or wellbeing of a member of their group | Facilitator/coach to contact Clinical Psychologist to discuss this further.  If follow-up required, Clinical Psychologist will contact the participant directly to assess risk severity and provide support in accordance with the “Risk Procedure for Research Staff” outlined below.  Clinical Psychologist will contact the GP if clinical assessment suggests that additional support is needed. |
| Participant reports an increase in symptoms or high dissociation in session | Participant can contact Clinical Psychologist directly to access further support, or the facilitator can do this on their behalf.  An increase in symptoms is not uncommon when beginning a trauma-focussed intervention. Clinical Psychologist will normalise the experience, provide psychoeducation on what happens when avoidance is removed, and assist participant to identify coping strategies (including grounding if dissociation is high) and existing supports.  If any risk is identified, Clinical Psychologist will assess risk severity and provide support in accordance with the “Risk Procedure for Research Staff” outlined below. |
| Participant expresses concerns about the wellbeing of a peer | Concerned participants are encouraged to discuss this with the facilitator/coach, who can contact Clinical Psychologist directly to discuss this further.  If follow-up required, Clinical Psychologist will contact the participant directly to assess risk severity and provide support in accordance with the “Risk Procedure for Research Staff” outlined below. |
| If a risk incident occurs in a group session | Facilitator to work to secure safety of all participants.  If there is an immediate threat or emergency, contact emergency services (000) immediately.  One facilitator to remove the individual(s) involved in the incident from the group if necessary to de-escalate the situation. Other facilitator to stay with the group.  Report incident to research team via duty phone as soon as possible.  Clinical Psychologist to inform GP, conduct a debriefing session with all affected individuals and provide support resources to those impacted. |
| If someone is presenting drug and alcohol effects | If a participant appears to be under the influence of drugs or alcohol, immediately assess the situation for safety concerns.  Ensure that the participant is not posing a risk to themselves or others.  If there is an immediate threat or emergency, contact emergency services (000) immediately.  If no immediate risk, facilitator to call a taxi to take the participant home, and if participant is unstable, contact their emergency contact person. If instability is high, emergency contact person will be asked to come and collect the participant, rather than sending them home in a taxi.  Offer the participant a safe and private space to rest and recover if needed and ensure they are supervised.  Report incident to research team as soon as possible.  If the participant is experiencing severe symptoms or a medical emergency, seek professional medical help immediately. |
| If a participant has taken action to harm themselves or others | Implement emergency procedures:   - Call 000 - Take necessary steps to ensure safety (e.g. apply First Aid or ensure safety of their environment) - Remain with the individual until help arrives - Report incident to research team as soon as possible.   Clinical Psychologist to inform GP, conduct a debriefing session with all affected individuals and provide support resources to those impacted. |

#### Risk Assessments

All risk assessments following a notice of concern from participants or facilitators will be conducted by a Clinical Psychologist and will be guided by the risk assessment guides attached below. It is not the role of facilitators to complete risk assessments or safety planning. Instead, facilitators can provide more general emotional support, remind them to draw upon strategies that have worked in the past, and provide crisis numbers (1800 RESPECT, Lifeline), as an interim measure prior to the Clinical Psychologist contacting the participant.

#### Documentation

Risk procedures for program facilitators are clearly outlined in the treatment manual provided to all facilitators/coaches of both the LWH and boxing-only programs.

Suicide risk will be carefully documented by the Clinical Psychologist in the encrypted, password-protected “Clinical” folder on OneDrive. These records are only accessible to Clinical Psychologist and Lead Researcher to determine appropriate next steps. In the occurrence of an adverse event, these records may be released to the Clinical Governance team at the University of Melbourne. Participants have been informed of limits of confidentiality in the Plain Language Summary and Consent Form.

#### Reporting of risk incidents

As per the protocol, the Trial Steering Committee (TSC) will be notified of all potential risk incidents.

The TSC will determine suitability of continuing the trial (i.e., determine stopping), along with reporting to the ethics committee, Clinical Governance, and trials registry. If reports are deemed necessary, these will be made by the Trial Management Committee.

Risks that are classified as extreme or high will be dealt with immediately; risks that are medium will be dealt with by the next Management Committee meeting, risks that are low will be monitored.

#### Follow-up

Clinical Psychologist will follow-up with participant and/or facilitators following an incident to check-in and offer additional support where required.

*Crisis Assessment Treatment Team Contact Numbers*

If an individual is expressing intent to harm themselves, a clinically trained member of research team will contact a Crisis Assessment Treatment Team (CATT). CATT operates on a catchment model, with different contact numbers for each catchment area. The area service for the individual’s actual location at the time should be contacted. Numbers for program locations have been outlined below. Should the risk arise outside of these areas (i.e. when the participant is at home), please review this website to find the appropriate number for the relevant suburb: <http://www3.health.vic.gov.au/mentalhealthservices/>

Please note the numbers provided below are for adults aged 18 to 65. For a participant outside this age group, please refer to the above website and select the relevant age bracket next to the suburb listing.

**Thoughts of death**

‘Are you feeling as though life isn’t worth living?’

‘Have you had any thoughts about harming yourself this week?’

**Ideation**

‘Have you felt like acting on these thoughts?’

‘Have you considered actually ending your life?’

**Yes**

**Yes**

**No**

**No**

**Yes, Intent**

**No**

**Intent**

**Intent**

‘Have you made any plans about how you would do this?’

‘Do you intend to act on this plan?’

**If you feel that intent may easily change, treat as high risk**

**Moderate risk**

- Explain that you would like to help them to keep feeling like they don’t need to use their plan to end their life

- Engage participant in grounding activity

- Remind them of self-care plan discussed at intake. Encourage implementation of plan and assist with problem-solving around any potential barriers to implementation.

- Encourage them to reach out to existing mental health supports

- Discuss next steps such as when they are scheduled to next see their MH support provider, what they plan to do between now and then to keep themselves safe, available supports in the interim (e.g. Lifeline, 1800RESPECT)

**Low risk**

- Engage participant in grounding activity

- Remind them of self-care plan discussed at intake. Encourage implementation of plan and assist with problem-solving around any potential barriers to implementation.

- Encourage them to reach out to available supports (existing mental health supports, Lifeline, 1800RESPECT)

**High risk**

- Express how hard and stressful it must be for them. Tell them that it is your job to support them and help them stay safe.

- Support the participant to create a more formalised safety plan using template attached below.

- If, after appropriate assessment, participant is deemed at immediate risk of harming themselves or others, they will be supported to seek support from CAT services or, 000.

- If participants are not willing or unable to contact services themselves, they will be advised that we must act on their behalf without their consent. Psychiatric support will be enlisted from CAT services or 000 on behalf.

- Once appropriate steps have been taken to ensure the participant’s immediate safety. participants regular mental health provider will be advised of risk and steps taken as soon as possible.

**Thoughts of death**

‘Are you feeling as though life isn’t worth living?’

‘Have you had any thoughts about harming yourself this week?’

**Ideation**

‘Have you felt like acting on these thoughts?’

‘Have you considered actually ending your life?’

**Yes**

**Yes**

**No**

**No**

**Yes, Intent**

**No**

**Intent**

**Intent**

‘Have you made any plans about how you would do this?’

‘Do you intend to act on this plan?’

**If you feel that intent may easily change, treat as high risk**

**Low risk**

- Many trauma survivors may experience ideation but not have any current plans to act on this

- Encourage them to reach out to available supports (existing mental health supports, reaching out to clinical psychologist, Lifeline, 1800RESPECT)

**Moderate risk**

- Be calm, non-judgemental and empathic

- Allow as much time as needed for disclosure and containing

- Explain that you would like to help them to keep feeling like they don’t need to use their plan, and that it is policy that the clinical psychologist will contact them to check in and help them get support.

-Inform clinical psychologist after you finish the session so they can follow up with a phone call

**High risk**

- Be calm, non-judgemental and empathic; allow as much time as needed

- Explain to the that it is your job to help them stay safe, which means you will need to talk to the clinical psychologist

- Finish your session while you wait for a clinician to call back. If finished, sit with them, offer them a cup of tea. Don’t discuss suicide risk, the clinician will take care of this.

- Before they leave, please make sure you have spoken to a clinician and made arrangements that you believe will protect them from harm, no matter how long it takes.

- If they refuse input we may have to act without their consent, but all participants are aware of this, and the clinician will assess this need.

**References for Supplementary Materials**

1. Schwarzer R, Jerusalem M, Weinman J, Wright S, Johnston M. Generalized Self-Efficacy Scale. Measures in Health Psychology: A User’s Portfolio Causal and control beliefs Windsor. 1995;

2. Cloitre M, Shevlin M, Brewin CR, Bisson JI, Roberts NP, Maercker A, et al. The International Trauma Questionnaire: development of a self‐report measure of ICD‐11 PTSD and complex PTSD. Acta Psychiatr Scand. 2018;138:536–46.

3. Meiser-Stedman R, Smith P, Yule W, Dalgleish T. The Trauma Memory Quality Questionnaire: Preliminary development and validation of a measure of trauma memory characteristics for children and adolescents. Memory. 2007;15:271–9.

4. Foa EB, Ehlers A, Clark DM, Tolin DF, Orsillo SM. The Posttraumatic Cognitions Inventory (PTCI): Development and validation. Psychological Assessment. 1999;11:303–14.

5. Henry JD, Crawford JR. The short-form version of the Depression Anxiety Stress Scales (DASS-21): Construct validity and normative data in a large non-clinical sample. British Journal of Clinical Psychology. 2005;44:227–39.

6. Russell D, Peplau LA, Cutrona CE. The revised UCLA Loneliness Scale: Concurrent and discriminant validity evidence. Journal of Personality and Social Psychology. 1980;39:472–80.

7. Lee RM, Robbins SB. Measuring belongingness: The Social Connectedness and the Social Assurance scales. Journal of Counseling Psychology. 1995;42:232–41.

8. Stewart-Brown S, Tennant A, Tennant R, Platt S, Parkinson J, Weich S. Internal construct validity of the Warwick-Edinburgh Mental Well-being Scale (WEMWBS): a Rasch analysis using data from the Scottish Health Education Population Survey. Health Qual Life Outcomes. 2009;7:15.

9. Hawthorne G. Assessing utility where short measures are required: development of the short assessment of Quality of Life-8 (AQoL-8) instrument. Value Health. 2009;12:948–57.

10. Saunders JB, Aasland OG, Babor TF, De La Fuente JR, Grant M. Development of the Alcohol Use Disorders Identification Test (AUDIT): WHO collaborative project on early detection of persons with harmful alcohol consumption-II. Addiction. 1993;88:791–804.

11. Morin CM, Belleville G, Bélanger L, Ivers H. The Insomnia Severity Index: psychometric indicators to detect insomnia cases and evaluate treatment response. Sleep. 2011;34:601–8.

12. Cleeland C S. Brief Pain Inventory User Guide. 2009.
