# Supplementary material for: Left Write Hook: trial protocol for a community-based type II hybrid effectiveness-implementation cluster randomised controlled trial of a boxing and writing-based intervention for adult survivors of child sexual abuse and gender-based violence in Australia
Source: BMJ Open. 2025 Sep 10;15(9):e099536. doi: 10.1136/bmjopen-2025-099536 (PMC12519368; doi:10.1136/bmjopen-2025-099536)
Supplement: Supplementary file 2 [file bmjopen-2025-099536.reviewer_comments.pdf]

PEER REVIEW HISTORY

BMJ Open publishes all reviews undertaken for accepted manuscripts. Reviewers are asked to complete a checklist review form and are provided with free text boxes to elaborate on their assessment. These free text comments are reproduced below.

ARTICLE DETAILS

Title (Provisional)

Left, Write, Hook: Trial Protocol for a Community-Based Type II Hybrid Effectiveness-Implementation Cluster Randomised Control Trial of a Boxing and Writing-Based Intervention for Adult Survivors of Child Sexual Abuse and Gender-Based Violence in Australia

Authors

Butler, Molly; Veltman, Emma; Lyon, Donna; Koye, Digsu Negese N; Fitzpatrick, Phoebe; Alisic, Eva; Ney, Luke; Goode, Anna; Healy, Genevieve; Hitchcock, Caitlin

---

VERSION 1 - REVIEW

**Reviewer**	**1**
**Name**	**Pebole, Michelle M.**
**Affiliation**	**Veterans Affairs Boston Healthcare System**
**Date**	**09-Jun-2025**
**COI**	**None**

Thanks for the opportunity to review this interesting and important paper, a protocol piece focused on left, write, hook: a survivor-led and trauma-informed boxing intervention. This is an important topic and intervention which aims to improve health among an important community. I believe this intervention can contribute to health promotion among women survivors. However, there are several points that the authors may consider revising to enhance transparency, clarity, and rigor. I offer these suggestions, in hopes that the authors agree that addressing will improve the manuscript. Detailed comments and suggestions are below.

The authors often shift between using the terms sexual violence, sexual abuse, and gender-based violence throughout the manuscript, and do not indicate what is included in these terms. Definitions of these terms are often different between countries – and many lay readers may not fully understand the differences between these terms. Thus, it is important for the authors to provide clear definitions or explanations throughout the paper, and to use consistent terminology throughout the manuscript. For example, I am unsure what exactly child sexual abuse and / or other gendered violence includes? Does this include family violence. What about childhood emotional abuse? It is true that children can experience childhood gendered abuse that is not sexual. It is possible that the authors are meaning to be inclusive of these forms of violence, but without a clear definition and consistent terminology it is hard to understand what they are referring to.

Pg 8 line 28 – 32. The authors note that they intend to evaluate an intervention by increasing self-efficacy and say that self-efficacy can improve physical and mental health. It is my view that this section should be supported with much more evidence, given that the authors identify it is as main mechanism of the intervention, and assert that improving self-efficacy can improve physical and mental health. Currently, they do not provide sufficient references to support these claims. It is especially important to provide this information, as well as directly connect self-efficacy to exercise for the reader, given that it is identified as the primary outcome of this trial.

Pg 8 – last sentence. Consider softening the language from "will" to "can"

Relatedly, the authors should add a citation after Pg 10, lines 3-10: "Collectively, current research suggests that a program that incorporates both a physical health component (e.g., exercise) with psychological component (e.g. build selfefficacy, promote processing of trauma-related memories and meaning) could help to effectively drive behaviour change in this high-risk population." Can the authors cite studies supporting this. A couple of example studies which may be worthwhile to include here are: exercise coupled with trauma-focused therapy (like prolonged exposure) and mental health groups (like survivor-centered group therapy) improves health outcomes. There is also good evidence for written trauma treatments like written exposure therapy (WET). Adding in citations to these bodies of work may strengthen the authors arguments for the importance and evidence-base of this intervention.

The authors then go on to talk about evidence from the pilot study of left, write, hook. While it is encouraging to see these beneficial outcomes, it does leave the reader with questions. Specifically, without some type of explanation, it is unclear how the intervention "left, write, hook" targets self-efficacy and why that was chosen as the primary outcome for the intervention. Especially given that self efficacy is not mentioned as a notable result in the introduction.

Additionally, the mechanism of the boxing to improve self efficacy is left unclear. It is possible that the interventionists support for self-efficacy throughout the intervention, but that is never really explained. As of now, I am wondering how to connect the dots between the intervention and self-efficacy, and I am also wondering how and why the authors chose self-efficacy as their primary outcome for this intervention.

It seems that more information about the pilot study would be beneficial. More information about all the measured outcomes, definitions of acceptability and feasibility, and themes would be beneficial. A mention of feasibility and acceptability measures are especially relevant, given that this manuscript and project focus on implementation. In my view, this context is extremely important and should be expanded in the introduction. Additionally, it is still unclear if and how the pilot data impacted self-efficacy, and how self-efficacy was chosen as the outcome for this version of the intervention.

In general, did this team consider co-occurring physical violence (e.g., traumatic brain injury, which is extremely prevalent in GBV and impacts intervention outcomes and adherence)?

The authors did not set up the reasons for comparing left write hook to a trauma informed boxing only intervention. Why not compare to a "usual care" (or some other control) control group. It seems that this intervention is primarily examining augmenting an established boxing intervention with expressive writing. If this is the case, it is not really fully understood until the last sentence of the introduction. Additionally, if this is the case, the authors have not provided

enough evidence to indicate that expressive writing will meaningfully improve the intervention (see above comment about references). It would benefit the manuscript to frame the intervention this way from the start, if this assumption is true. If not, explaining the use of the control group would be beneficial.

The focus of the manuscript is confusing, and I believe the introduction should give more background / and introduction to the implementation focus of this project. At the end of the introduction, the reader is led to believe the focus of this trial is comparing self-efficacy between LWH and TI Boxing. However, in the study design section, the authors indicate the primary outcome is implementation - fidelity of the intervention training and delivery. The manuscript needs substantial work to set up implementation outcomes and their relevance for this project in the introduction section. As written, the implementation part seems like it comes out of nowhere to the reader.

Additionally, it is never fully explained how this intervention is trauma-informed, only that it is trauma -informed. Given that trauma informed care in exercise settings is new and that delivery of trauma informed exercise can vary dramatically from place to place, it would be beneficial to describe several strategies as to how LWH and the control intervention are trauma informed.

Inclusion criteria: women must be willing to "provide contact details for their GP to ensure that participants can safely participate" – can the authors provide more information about how they assessed if survivors could safely participate? Does this mean physical safety – or does this refer to psychological safety (e.g., ongoing violence). Regarding trauma-informed care, the authors cover inclusion / exclusion criteria. Were there any inclusion / exclusion criteria related to ongoing violence that may be happening ? This seems like an essential consideration that has also been documented in some exercise research for survivors of sexual and gender based violence. If so, this should be mentioned. If not, this should be discussed – or at least mentioned.

Authors state that LWH costs up to $450 dollars when accessed in the community (pg 13). Is there already evidence for the efficacy of LWH (I guess so, otherwise how would it have been implemented at this cost in the community?). This seems like a really important piece to explain to the reader at the start of the manuscript. If this study is focused on an already evidence-based intervention, what new data and results will it bring? Is it focused on another outcome, or does it involve something new?

Can the authors provide evidence for "expressive writing" and how / if it is effective on its own? Can they provide evidence that the boxing intervention was trauma informed?

Based on the authors description of the control group, I am not convinced that the control group is trauma informed, rather just a boxing class. Can the authors provide evidence that this group is trauma – informed? The fact that it is not peer led already changes the dynamic of the boxing class from the LWH boxing class. So, it seems the goal of this intervention may not have been to augment trauma informed boxing, rather to test two separate interventions (LWH, cardio boxing) to each other. Can the authors clarify? Can the authors provide rationale as to why they used this group as the control group?

Pg 17 - authors say this intervention is meant to "empower adult survivors of sexual violence." Per my first point, is this true? What about other forms of gender based violence? Who is included in this intervention?

Pg 27 – the authors say low technology means they can implement the program in remote communities – some explanation is needed there, as remote interventions can sometimes be more accessible for remote or rural dwelling people – if these communities have internet access, given that they wouldn't have to drive to an exercise setting which may be far away. Perhaps the authors could provide more information or clarify this line of thought.

Again, I thank the authors for implementing this thoughtful and important project to serve women experiencing violence. I hope the authors agree that addressing these points can improve the clarity and transparency of this project, and provide helpful details which will be useful for other researchers who would like to implement interventions to improve health among these communities. I wish the authors well with these revisions.

**Reviewer**	**2**
**Name**	**Rosenbaum, Simon**
**Affiliation**	**University of New South Wales**
**Date**	**11-Jun-2025**
**COI**	**None**

General Comments

This is a timely and innovative protocol addressing the critical need for accessible, trauma-informed physical activity interventions for survivors of gender-based violence. I have made a number of comments which hopefully help to further strengthen the manuscript. Things to consider include providing greater conceptual clarity, particularly regarding definitions, intervention distinctions, and the rationale for the trial design. Strengthening the articulation of trauma-informed care principles and the underlying evidence base may help improve overall coherence and translational value of the protocol.

Abstract

1. The rationale for comparing trauma-informed boxing plus expressive writing to boxing alone needs to be more clearly stated. What gap in the literature is this study addressing?

2. It would help to clarify early on whether both arms are trauma-informed, as this is only implied.

3. Consider rephrasing "these individuals" (p.6, line 16).

Background

1. Please define the target population clearly and consistently. The protocol uses multiple terms (e.g., sexual violence, gendered violence, child sexual abuse, family violence) without clarifying whether these are interchangeable or overlapping.

2. The concept of trauma-informed care is mentioned but not clearly defined. An early explanation of key principles would be useful, especially for readers unfamiliar with the framework.

3. On page 9 (line 16), consider rephrasing “engaging in health risk behaviours” and provide specific examples relevant to survivors.

4. When referring to boxing (lines 17, 22), please clarify whether this is “non-contact boxing” and use consistent terminology throughout. This distinction is important given the nature of the intervention and population.

5. The discussion of peer-led approaches needs further justification. The idea that support can be delivered by someone without formal clinical training is an important one but requires clearer explanation and referencing (see also p.10, line 38).

6. It would be helpful to clarify whether “champions,” “peer-led,” and “survivor instructors” are synonymous or distinct roles.

7. Provide clearer justification for the focus on peer-led delivery, the theoretical and practical value is implied but not explicitly stated.

8. A brief overview of the evidence gaps that the RCT will address is needed before the aims section. This will help contextualize the decision to compare the two intervention arms.

9. Consider whether the trial should be framed as a hybrid effectiveness-implementation design. The current wording suggests the aim is to “develop an implementation strategy,” but there may be scope to more explicitly evaluate both implementation outcomes (e.g., fidelity) and participant-level outcomes (e.g., self-efficacy).

Methods

1. The process for obtaining GP clearance is unclear. Would asking participants to provide a clearance letter from their GP introduce unnecessary barriers, especially for those with histories of trauma or limited access to health care? Could a validated screening tool (e.g., the ESSA Adult Pre-Exercise Screening Tool) offer a more inclusive and efficient option?

2. Clarify whether a “history of abuse” includes current experiences of abuse, this has ethical and safety implications for inclusion and risk management.

3. The term “safely participate” (p.13, line 55) could be unpacked. If this refers to psychological safety rather than physical risk, it would be helpful to clarify how the intervention delivery fosters a trauma- and violence-informed sense of safety. Consider describing specific mechanisms or processes that contribute to this (e.g., control of space, consent practices, facilitator behaviour).

4. In the intervention description (p.15), it is unclear if the boxing delivered in the control arm differs in key ways (e.g., peer-led, empowerment focus). If so, this could confound interpretation, is any observed effect due to expressive writing or to the peer-led model?

5. Please specify which hormone(s) (e.g., cortisol) are being assessed.

6. Trauma- and violence-informed principles are described as underpinning the intervention, but this needs clearer articulation. A table or diagram outlining how these principles are embedded in each arm would improve clarity.

7. The movement behaviour assessment (Table 2) requires a reference to support its reliability and validity.

8. Consider including the risk management protocol as an appendix, given the vulnerability of the target group.

9. Clarify your approach to positionality. Given the survivor-led and co-designed nature of the intervention, it would be appropriate to reflect briefly on the research team's perspectives and positionalities (p.18).

10. The advisory group is comprised of CSA survivors, but the intervention appears broader in scope. Would it be appropriate to include survivors of other forms of gendered violence?

11. Page 22, line 31: Please elaborate on the qualitative analysis approach. Thematic analysis is mentioned, but a brief description or reference (e.g., Reflexive Thematic Analysis) would enhance transparency.

Discussion

1. The case for peer-led delivery (p.27) needs to be better established earlier in the paper. Also, while the model may be low-cost, consider the implications of equitable pay for peer facilitators. The term "peer-led" should be clearly defined. Are peer facilitators also survivors? What support structures are in place?

2. The term "trauma processing" (p.27, line 22) may not be accurate when describing sharing trauma narratives in group settings. Consider clarifying whether this is about expression, validation, or meaning-making.

3. The support structures for peer facilitators (mentioned p.28, line 38) are not well described in the methods. If available, this detail should be included.

VERSION 1 - AUTHOR RESPONSE

Reviewer 1: Dr. Michelle M. Pebole

Thanks for the opportunity to review this interesting and important paper, a protocol piece focused on left, write, hook: a survivor-led and trauma-informed boxing intervention. This is an important topic and intervention which aims to improve health among an important community. I believe this intervention can contribute to health promotion among women survivors. However, there are several points that the authors may consider revising to enhance transparency, clarity, and rigor. I offer these suggestions, in hopes that the authors agree that addressing will improve the manuscript. Detailed comments and suggestions are below.

1. The authors often shift between using the terms sexual violence, sexual abuse, and gender-based violence throughout the manuscript, and do not indicate what is included in these terms. Definitions of these terms are often different between countries – and many lay readers may not fully understand the differences between these terms. Thus, it is important for the authors to provide clear definitions or explanations throughout the paper, and to use consistent terminology throughout the manuscript. For example, I am unsure what exactly child sexual abuse and / or other gendered violence includes? Does this include family violence. What about childhood emotional abuse? It is true that children can experience childhood gendered abuse that is not sexual. It is possible that the authors are meaning to be inclusive of these forms of violence, but without a clear definition and consistent terminology it is hard to understand what they are referring to.

Author response: Thank you for this comment regarding the clarity of terminology used throughout the manuscript. We have revised our terminology to consistently use the term gender-based violence, per the Australian government's definition. This term is inclusive of any experience of sexual violence, or any physical, sexual, or emotional violence from an intimate partner or family member, in either adulthood or childhood. Addressing gender-based violence is a national priority for the Australian government (who funded this research). Our lived experience researchers stressed the importance of creating space in our inclusion criteria for individuals to self-identify with these terms, rather than have the research team determine whether their experiences met a particular threshold for violence or abuse, to uphold principles of accessibility and agency. We have added a definition of gender-based violence to the Methods (pg 17).

Pg 17: 'For the purposes of this study, the term 'gender-based violence' is inclusive of any experience of sexual violence, or any physical, sexual, or emotional violence from an intimate partner or family member, in either adulthood or childhood.'

2. Pg 8 line 28 – 32. The authors note that they intend to evaluate an intervention by increasing self-efficacy and say that self-efficacy can improve physical and mental health. It is my view that this section should be supported with much more evidence, given that the authors identify it is as main mechanism of the intervention, and assert that improving self-efficacy can improve physical and mental health. Currently, they do not provide sufficient references to support these claims. It is especially important to provide this information, as well as directly connect self- efficacy to exercise for the reader, given that it is identified as the primary outcome of this trial.

Author response: This is an important point, thank you.

Additional information with evidence to support selection of self-efficacy as the primary outcome has been provided on pages 13-14 of the manuscript (under the heading 'Self-Efficacy').

Pg 13-14: 'There are multiple processes of change which may drive improvements in both mental and physical health, and our pilot data suggested that Left Write Hook may exert its

effects by empowering survivors. Here, we evaluated program effects on self-efficacy for a number of reasons. First, the General Self Efficacy Scale [53] has strong psychometric properties and incorporates elements of agency, assertiveness and control to assess individual's belief in their overall capability to succeed—factors which all improved in our pilot data. Self-efficacy is persistently low in survivors, reflecting pervasive perceptions of a lack of self-agency and power over their own lives [54]. Similarly, survivors may mentally disconnect from their bodies (i.e., dissociate) in order to cope with their experiences and can as a result feel a lack of agency over their own bodies [55]. Critically, self-efficacy is longitudinally associated with mental and physical health outcomes in both community and trauma-exposed populations, with greater self-efficacy linked to decreases in PTSD symptoms, self-report pain, fatigue and substance use, alongside increases to physical activity [56,57]. Finally, aligned with our co-production approach, survivors were central to deciding the primary outcome for this trial. Our lived experience advisors stressed the importance of an outcome measure that is recovery oriented rather than symptom based, which reflects how the survivor is moving forward with their life, rather than emphasising ongoing challenges. We therefore considered intervention efficacy on self-efficacy, with secondary outcomes of mental and physical health.'

3. Pg 8 – last sentence. Consider softening the language from "will" to "can"

Author response: Thank you for picking this up. This line has been removed.

4. Relatedly, the authors should add a citation after Pg 10, lines 3-10: "Collectively, current research suggests that a program that incorporates both a physical health component (e.g., exercise) with psychological component (e.g. build self efficacy, promote processing of trauma- related memories and meaning) could help to effectively drive behaviour change in this high-risk population." Can the authors cite studies supporting this. A couple of example studies which may be worthwhile to include here are: exercise coupled with trauma-focused therapy (like prolonged exposure) and mental health groups (like survivor-centered group therapy) improves health outcomes. There is also good evidence for written trauma treatments like written exposure therapy (WET). Adding in citations to these bodies of work may strengthen the authors arguments for the importance and evidence-base of this intervention.

Author response: Thank you for your generosity in guiding us on how to strengthen our the rationale for Left Write Hook. Additional evidence has been added under the subheadings 'Writing-Based Interventions' (pg 10) and 'Body-Based Interventions' (pg 11). Notably, evidence has been added to support our claims that physical (i.e. non-contact boxing) and psychological (i.e. expressive writing, written exposure therapy) treatment components may be particularly effective in combination.

Pg 10, Writing-Based Interventions: 'There is a strong evidence base for the efficacy of writing-based interventions such as written exposure therapy [30–32], wherein survivors

write expressively about the trauma with the guidance of a trained support person [30]. As written-based therapeutic interventions require less specialist training and clinical expertise to deliver relative to current first-line treatments (e.g., EMDR) [14], they are well suited to peer delivery. Aligned with current first-line treatments, writing-based therapies facilitate the cognitive processing of trauma-related schema [33,34] while developing capacity to tolerate and process difficult internal experiences [32]. Writing about trauma in a supportive peer environment may strengthen survivors' coping skills by teaching them that their trauma-memories are not dangerous, that distress associated with recalling trauma is both tolerable and transient, and that it is possible to develop new perspectives on trauma and its meaning [30]. In addition to established effects for psychopathology including reductions in symptoms of posttraumatic stress disorder (PTSD), anxiety and depression [35–37], there are some suggestions that expressive writing is associated with improvements for both objectively assessed and self-reported physical health outcomes [38]. While writing offers valuable benefits for trauma processing [33,34], an important therapeutic goal for survivors is to reconnect with and regain agency over their bodies [39]. Moreover, recent evidence has demonstrated that augmenting trauma-focused exposure therapy with aerobic exercise can improve therapeutic outcomes [40]. Accordingly, incorporating embodied practices that help survivors ground themselves in the present and reconnect with their physical self after engaging with trauma memories through writing may therefore enhance therapeutic effects.'

Pg 11, Body-Based Interventions: 'Integrating body-based and cognitive techniques may better address survivors' diverse needs and promote sustained recovery outcomes [41,42]. There are a number of body-based therapeutic techniques which are beneficial for trauma survivors. In particular, non-contact boxing offers promise as a modality to help survivors reconnect with their bodies and ground into the present Non-contact boxing combines high intensity training and bodily awareness through techniques including stretching, skipping, shadowboxing, pad work, and bag work [43]. It is shown to foster an enhanced sense of empowerment and ability to locate healthy aggression in survivors [44], along with increases in agency, resilience, self-esteem and perceived physical ability [45]. Encouragingly, exercise-based adjuncts fit easily into peer-delivery models and have demonstrated impacts for survivors in improving symptoms of PTSD, depression and anxiety [43]. Improvements extend beyond mental health, with evidence of physical activity interventions reducing risk of chronic disease (including cardiovascular, respiratory, and type 2 diabetes) by up to 80% . Given potential benefits for improving both mental and physical health in survivors, combining this physiological-based approach with an established psychological technique such as expressive writing may help to enhance outcomes for survivors.'

5. The authors then go on to talk about evidence from the pilot study of left, write, hook. While it is encouraging to see these beneficial outcomes, it does leave the reader with questions. Specifically, without some type of explanation, it is unclear how the intervention "left, write, hook" targets self-efficacy and why that was chosen as the primary outcome for the intervention. Especially given that self-efficacy is not mentioned as a notable result in the introduction.

Additionally, the mechanism of the boxing to improve self-efficacy is left unclear. It is possible that the interventionists support for self-efficacy throughout the intervention, but that is never really explained. As of now, I am wondering how to connect the dots between the intervention and self-efficacy, and I am also wondering how and why the authors chose self-efficacy as their primary outcome for this intervention.

Author response: It was an oversight on our part, not to mention self-efficacy in the Background. We now include a subsection 'Self-Efficacy' justifying our choice of primary outcome (pg 13-14, excerpt included in response to comment 2). We also detail the evidence for an effect of non-contact boxing on self-efficacy (pg 11);

Pg 11: 'Non-contact boxing combines high intensity training and bodily awareness through techniques including stretching, skipping, shadowboxing, pad work, and bag work [43]. It is shown to foster an enhanced sense of empowerment and ability to locate healthy aggression in survivors [44], along with increases in agency, resilience, self-esteem and perceived physical ability [45].'

6. It seems that more information about the pilot study would be beneficial. More information about all the measured outcomes, definitions of acceptability and feasibility, and themes would be beneficial. A mention of feasibility and acceptability measures are especially relevant, given that this manuscript and project focus on implementation. In my view, this context is extremely important and should be expanded in the introduction. Additionally, it is still unclear if and how the pilot data impacted self-efficacy, and how self-efficacy was chosen as the outcome for this version of the intervention.

Author response: Thank you for highlighting the need for more explanation regarding the pilot study. Additional information has been added to the manuscript to clarify the outcome measures from the pilot study and how the results supported progression to a randomised controlled trial.

Pg 12-13: 'In our pilot study with adult survivors of child sexual abuse, large effect sizes were seen for pre- to-post intervention improvement on a self-report measure of emotional, social, and psychological wellbeing (Cohen's d=0.99), alongside clinically meaningful reductions in PTSD symptomatology (Cohen's d=1.02) [52]. Survivors who completed the program experienced a large increase (Cohen's d=1.48) in the self-reported ability to assert oneself, affect one's environment, and perform actions [52]. This increased self-efficacy was reflected in qualitative analysis which demonstrated that the expressive writing promoted themes of participant empowerment, validation and connection to one's body (e.g., 'I want to fix my body and my mind and feel strong'), with writing outputs reflecting that these themes evolved positively across the course of participation. Demonstrating feasibility and acceptability, validation of participants' stories and feelings also emerged as a key qualitative theme, and 80% of participants completed all sessions and research measures. Formal evaluation of program efficacy, acceptability, and feasibility is now needed.'

Pg 14: 'Finally, aligned with our co-production approach, survivors were central to deciding the primary outcome for this trial. Our lived experience advisors stressed the importance of an outcome measure that is recovery oriented rather than symptom based, which reflects how the survivor is moving forward with their life, rather than emphasising ongoing challenges. We therefore considered intervention efficacy on self-efficacy, with secondary outcomes of mental and physical health.'

The reviewer may be interested to know that we currently have submitted elsewhere an uncontrolled research trial of Left Write Hook. In this uncontrolled trial, we observed a significant decrease in self-report symptoms of depression, anxiety and stress, post-traumatic stress disorder, and complex post-traumatic stress disorder. We also observed a decrease in maladaptive post-traumatic cognitions and fragmentation of trauma memories, along with a significant increase in emotional wellbeing from pre- to post-intervention.

7. In general, did this team consider co-occurring physical violence (e.g., traumatic brain injury, which is extremely prevalent in GBV and impacts intervention outcomes and adherence)?

Author response: Thank you for raising this important consideration. Participants are asked to indicate any physical health conditions (such as Traumatic brain injury (TBI) or other cognitive impairment) at intake. We have added more detail on the intake process on page 17 under the heading 'Participant Recruitment'. Lived experience input during study design emphasised the importance of inclusivity, particularly with consideration to trauma-related health issues such as TBI, thus it was not listed as an exclusion criterion.

Pg. 17: 'All participants complete an intake interview with a clinical psychologist trained in risk formulation to collaboratively decide whether to participate. During the interview, the clinical psychologist queries physical health, mental health and situational factors (e.g. ongoing violence) that may impact an individual's safe participation'.

We have also added a line to the Discussion (pg 36) emphasising the need to consider effects of physical and/or chronic health conditions on intervention effects in future research;

Pg. 36: 'Similarly, it will be important to consider the influence of physical and/or chronic health conditions such as traumatic brain injury on any observed treatment effects, to indicate whether adjustments may need to be made for some survivors.'

8. The authors did not set up the reasons for comparing left write hook to a trauma informed boxing only intervention. Why not compare to a "usual care" (or some other control) control group. It seems that this intervention is primarily examining augmenting an established boxing intervention with expressive writing. If this is the case, it is not really fully understood until the last sentence of the introduction. Additionally, if this is the case, the authors have not provided enough evidence to indicate that expressive writing will meaningfully improve the intervention (see above comment about references). It would benefit the manuscript to

frame the intervention this way from the start, if this assumption is true. If not, explaining the use of the control group would be beneficial.

Author response: We appreciate you raising these concerns around the control group needing further clarification. Changes have been made throughout the 'Background' section to make improve the justification for the control group;

Pg 15: 'We compare Left Write Hook to a control group of trauma-informed non-contact boxing only, delivered by a personal trainer (rather than fellow survivor), akin to the type of current evidence-based program survivors can easily access in the community. The resource (i.e., both interventions able to be delivered by non-psychologists) and time commitment for participants was matched between trial arms.'

There is an existing evidence-base for the efficacy of non-contact boxing on both physical and mental health, which is now detailed on page 11 under the subheading 'Body-Based Interventions' (excerpt included in response to comment 4).

We have also further expanded the rationale for why Left Write Hook may have added benefits beyond non-contact boxing (pg 12).

Pg 12: 'Left Write Hook has potential to expand beyond the established benefits of non-contact boxing alone through three key enhancements: the peer facilitator model encourages social connection and belonging; the additional writing component enables individuals to reclaim their life narratives and process difficult internal experiences; and the empowerment focus carries themes from the writing through to the boxing components to encourage grounding into the self and expression through physical movement.'

Finally, further research would be required to isolate the exact therapeutic components of Left Write Hook, if the intervention were found to be effective. To reflect this, we have added a future direction of conducting a mechanisms trial to uncover the active ingredients of the intervention (pg 36);

Pg 36: 'A mechanistic approach may additionally be taken in the future to unpack the active ingredients of Left Write Hook, notably, the compounded effects of peer-delivery and the empowerment focus.'

9. The focus of the manuscript is confusing, and I believe the introduction should give more background / and introduction to the implementation focus of this project. At the end of the introduction, the reader is led to believe the focus of this trial is comparing self-efficacy between LWH and TI Boxing. However, in the study design section, the authors indicate the primary outcome is implementation - fidelity of the intervention training and delivery. The manuscript needs substantial work to set up implementation outcomes and their relevance for this project in the introduction section. As written, the implementation part seems like it comes out of nowhere to the reader.

Author response: Thank you for noting this. We have restructured the 'Background' section to make the rationale for the project clearer. In support of the implementation focus, we have added a section to justify our focus on implementation in the context of peer-delivery (pg 10) and have additionally added a section under the subheading 'Implementation' on the importance of fidelity of intervention training and delivery (pg 14).

Pg 10: 'Although growing evidence supports peer-led approaches for survivors, implementation challenges have hindered their adoption [27]. Thus, scaling peer-led interventions requires systematic evaluation of both effectiveness and implementation outcomes. Essential implementation components include developing comprehensive training protocols and establishing accessible supervision structures that enable peer facilitators to deliver interventions safely (for both themselves and those they support) and effectively [28,29].'

Pg 14: 'Beyond establishing treatment efficacy, considering implementation is essential for new interventions. A significant gap often develops when translating treatments developed within controlled research settings into real-world mental health settings [60]. Treatment fidelity, the degree to which interventions are delivered as intended, presents a fundamental challenge, as real-world delivery often deviates substantially from original protocols [61]. These challenges may be particularly pronounced in peer-delivery models, where facilitators have diverse professional backgrounds and experience [62]. Additionally, working with survivors of gender-based violence who often have complex and diverse needs may create tension between protocol adherence and dynamic, person-centred care. Maintaining intervention fidelity is essential not only to ensure treatments achieve their intended effects, but also to inform effective scaling to new contexts [61]. Therefore, systematically assessing fidelity across both training and delivery phases is fundamental to ensuring new interventions can be implemented safely and effectively in diverse settings [63]. Our use of a hybrid effectiveness-implementation trial design allows dual consideration of a primary effectiveness (individual-level) and primary implementation (individual level, site-level) outcomes [63], enabling formal evaluation of both potential benefits of the intervention for survivors, and the acceptability and feasibility of intervention delivery within established organisations (health services, charities) which support survivors.'

10. Additionally, it is never fully explained how this intervention is trauma-informed, only that it is trauma -informed. Given that trauma informed care in exercise settings is new and that delivery of trauma informed exercise can vary dramatically from place to place, it would be beneficial to describe several strategies as to how LWH and the control intervention are trauma informed.

Author response: This is a great point. We have included a table outlining how principles of trauma-informed care are embedded in each intervention, found on page 21-23 (Table 2)

11. Inclusion criteria: women must be willing to "provide contact details for their GP to ensure that participants can safely participate" – can the authors provide more information about how they assessed if survivors could safely participate? Does this mean physical safety – or does this refer to psychological safety (e.g., ongoing violence).

Author response: Additional information has been provided to define safe participation. We mean to refer to both psychological and physical safety.

Pg 18: 'Safe participation means that involvement in the program does not negatively impact an individual's physical or mental health, does not place them or others at risk of harm, and does not interfere with ongoing evidence-based treatments (e.g. participants undergoing EMDR may choose to delay participation until treatment is complete to avoid potential interference from the expressive writing component of Left Write Hook with memory reprocessing)'

Further information has also been added regarding how the research teams assessed safety to participate, which is outlined in response to comment 12.

12. Regarding trauma-informed care, the authors cover inclusion / exclusion criteria. Were there any inclusion / exclusion criteria related to ongoing violence that may be happening ? This seems like an essential consideration that has also been documented in some exercise research for survivors of sexual and gender based violence. If so, this should be mentioned. If not, this should be discussed – or at least mentioned.

Author response: Thank you for raising this important consideration. Our inclusion criteria are deliberately broad to create space for survivors to have a sense of agency over their participation, and to centre them as experts in their experiences and current situations. However, we have also carefully considered the implications of ongoing violence for participation. Our intake process involves collaborative decision making with a clinical psychologist who is trained to formulate risk, with a key focus being to establish physical and psychological safety. Where ongoing violence or other potential safety concerns are reported during the intake phone call, individuals are supported to make decisions about the best time for them to participate, with the focus being the prioritisation of their own safety and the safety of the group. When we speak about safety here, we seek to establish that involvement in the program will not negatively impact an individual's physical or mental health and does not place them or others at risk of harm. Where safety concerns are identified, the clinical psychologist will suggest delaying participation. Efforts will then be made to include the individual in later groups, once risk is minimised and the safety of participation can be established. This information is included in the manuscript on page 18 under the subheading 'Participant Recruitment'.

Pg 18: 'All participants complete an intake interview with a clinical psychologist trained in risk formulation to collaboratively decide whether to participate. During the interview, the clinical psychologist queries physical health, mental health and situational factors (e.g. ongoing violence) that may impact an individual's safe participation. Safe participation

means that involvement in the program does not negatively impact an individual's physical or mental health, does not place them or others at risk of harm, and does not interfere with ongoing evidence-based treatments (e.g. participants undergoing EMDR therapy may choose to delay participation until treatment is complete to avoid potential interference from the expressive writing component of Left Write Hook with memory reprocessing). Where safety concerns are identified, the clinical psychologist may recommend that the individual delay participation until their circumstances are more stable; for example, once an injury is healed, or external supports are in place. We initially pre-registered inclusion criteria to include a requirement for a letter of support from the GP, however decided to remove this to enhance equity of participation (i.e. in situations where the GP has been non-responsive). Instead, the participant will provide contact information for their GP and any other mental health professionals, with consent sought for them to be contacted if safety concerns arise in the intake interview or during the program. As per our risk management protocol (see supplementary materials for full protocol), if the trial team identify safety concerns, the GP or other health professionals will be contacted to confirm that the participant can take part safely and to ensure continuity of care. Where the GP or other health professionals involved in the person's care express concerns about safety of participation, this will be discussed with the individual so that they are aware of possible risks related to their involvement, and they will be invited to participate once their professional supports advise it is safe to do so.'

13. Authors state that LWH costs up to $450 dollars when accessed in the community (pg 13). Is there already evidence for the efficacy of LWH (I guess so, otherwise how would it have been implemented at this cost in the community?). This seems like a really important piece to explain to the reader at the start of the manuscript. If this study is focused on an already evidence- based intervention, what new data and results will it bring? Is it focused on another outcome, or does it involve something new?

Author response: Thank you for highlighting the need for clarification here. The Left Write Hook program has been developed via a bottom-up approach, whereby a survivor who identified a need for further support initiated a support group in their local community which has since grown to a charitable organisation which operates throughout Melbourne. As a result, this trial is unique as we are determining the efficacy of a program that already exists in the community. We have now further detailed the results from the prior pilot study to better illustrate the current evidence base, and edited the manuscript to clarify that the payment is a suggested charitable donation (pg 19).

Pg 19: 'Left Write Hook is currently accessible in the community (delivered via a survivor-led charity) through a 'pay what you feel' donation of up to $450 to the Left Write Hook charity.'

14. Can the authors provide evidence for "expressive writing" and how / if it is effective on its own?

Author response: Additional evidence for the effectiveness of expressive writing on improving self-efficacy and mental health outcomes has been added under the subheading 'Writing-Based Interventions' on page 10 (manuscript excerpt included in response to comment 4).

15. Can they provide evidence that the boxing intervention was trauma informed?

Author response: We have added a table that outlines how trauma-informed principles have been applied in the boxing-only group. Please see Table 2 on page 21.

16. Based on the authors description of the control group, I am not convinced that the control group is trauma informed, rather just a boxing class. Can the authors provide evidence that this group is trauma – informed? The fact that it is not peer led already changes the dynamic of the boxing class from the LWH boxing class. So, it seems the goal of this intervention may not have been to augment trauma informed boxing, rather to test two separate interventions (LWH, cardio boxing) to each other. Can the authors clarify? Can the authors provide rationale as to why they used this group as the control group?

Author response: Thank you for raising these points. As mentioned in response to comment 15, Table 2 now demonstrates how trauma-informed principles have been applied in the boxing-only group. The goal of this trial is to evaluate the effectiveness of the Left Write Hook intervention in comparison to trauma-informed exercise as usual, represented here by trauma-informed non-contact boxing. More detail on the choice of comparison condition has been added to both the Background (pg 15) and the Method (pg 23);

Pg 15: 'We compare Left Write Hook to a control group of trauma-informed non-contact boxing only, delivered by a personal trainer (rather than fellow survivor), akin to the type of current evidence-based program survivors can easily access in the community.'

Pg 23: 'Boxing was selected as the exercise modality based on consumer input that identified it as the most acceptable exercise option for an active control, particularly in the context of consenting to randomisation. The control group therefore represents trauma-informed exercise options as usual, without three unique components of the Left Write Hook intervention: the peer-led nature of intervention; expressive writing; and the focus on empowerment that links from the expressive writing to the trauma-informed non-contact boxing component to encourage expression through physical movement'

17. Pg 17 - authors say this intervention is meant to "empower adult survivors of sexual violence." Per my first point, is this true? What about other forms of gender based violence? Who is included in this intervention?

Author response: Thank you for noting this. We have changed the term sexual violence here to gender-based violence, and made similar changes throughout the manuscript to broaden

our terminology to 'gender-based violence'. Further detail has been added to clarify our inclusion criteria on page 17 under the subheading 'Participant Recruitment' (excerpt included in response to comment 1).

18. Pg 27 – the authors say low technology means they can implement the program in remote communities – some explanation is needed there, as remote interventions can sometimes be more accessible for remote or rural dwelling people – if these communities have internet access, given that they wouldn't have to drive to an exercise setting which may be far away. Perhaps the authors could provide more information or clarify this line of thought.

Author response: Thank you for picking this up. This line has been removed.

Again, I thank the authors for implementing this thoughtful and important project to serve women experiencing violence. I hope the authors agree that addressing these points can improve the clarity and transparency of this project, and provide helpful details which will be useful for other researchers who would like to implement interventions to improve health among these communities. I wish the authors well with these revisions.

Reviewer 2: Dr. Simon Rosenbaum

Comments to the Author:

This is a timely and innovative protocol addressing the critical need for accessible, trauma-informed physical activity interventions for survivors of gender-based violence. I have made a number of comments which hopefully help to further strengthen the manuscript. Things to consider include providing greater conceptual clarity, particularly regarding definitions, intervention distinctions, and the rationale for the trial design. Strengthening the articulation of trauma-informed care principles and the underlying evidence base may help improve overall coherence and translational value of the protocol.

Author response: Thank you for the positive feedback and suggested revisions. We have carefully addressed your feedback as outlined below.

Abstract

1. The rationale for comparing trauma-informed boxing plus expressive writing to boxing alone needs to be more clearly stated. What gap in the literature is this study addressing?

Author response: Further information has been added to the abstract to justify the rationale for our selection of control group. Please refer to page 5 or see below.

Pg 5: 'In this Type II hybrid effectiveness-implementation cluster randomised control trial, we evaluate a novel peer-led intervention that combines expressive writing and trauma-informed boxing, Left Write Hook, against trauma-informed boxing alone – an intervention

approach that is currently accessible in the community and has been shown to improve both mental and physical health.'

2. It would help to clarify early on whether both arms are trauma-informed, as this is only implied.

Author response: Thank you for noting this. We have specified that both arms are trauma-informed in the manuscript. We have also included a table outlining how principles of trauma-informed care are embedded in each arm (please see Table 2, pg 21).

3. Consider rephrasing "these individuals" (p.6, line 16).

Author response: We have changed this to 'creating space for empowerment' (pg 5).

Pg 5: 'Creating space for survivors to feel empowered through a multidimensional approach to health promotion, considering both physical and psychological influences on health, is necessary to reduce chronic disease.'

Background

1. Please define the target population clearly and consistently. The protocol uses multiple terms (e.g., sexual violence, gendered violence, child sexual abuse, family violence) without clarifying whether these are interchangeable or overlapping.

Author response: Thank you for noting these inconsistencies. The intervention is targeted at survivors of gender-based violence. For the purposes of this study, the term 'gender-based violence' is inclusive of any experience of sexual violence, or any physical, sexual, or emotional violence from an intimate partner or family member, in either adulthood or childhood. We have edited the manuscript to ensure consistency in our terminology and to include this exact definition (pg 17).

Pg 17: 'For the purposes of this study, the term 'gender-based violence' is inclusive of any experience of sexual violence, or any physical, sexual, or emotional violence from an intimate partner or family member, in either adulthood or childhood.'

As the Left Write Hook intervention was initially designed for survivors of child sexual abuse (CSA), we received input from the charity that specifically naming CSA is important. This is why CSA is sometimes mentioned specifically alongside mention of gender-based violence (e.g. in our inclusion criteria).

2. The concept of trauma-informed care is mentioned but not clearly defined. An early explanation of key principles would be useful, especially for readers unfamiliar with the framework.

Author response: Thank you for this suggestion. A definition of trauma-informed care has been included on page 9 under the subheading 'Trauma-Informed, Peer-Led Interventions'.

Pg 9: 'Trauma-informed care encompasses principles of safety; trustworthiness and transparency; peer support; collaboration and mutuality; empowerment, voice, and choice; and cultural, historical, and gender issues [23].'

3. On page 9 (line 16), consider rephrasing "engaging in health risk behaviours" and provide specific examples relevant to survivors.

Author response: Thank you for picking up on this. The wording has been changed on page 8.

Pg 8: 'Survivors have higher levels of stress which can increase engagement in health risk behaviours such as reduced physical activity, substance use and unhealthy diet [7], each of which uniquely contribute to the development of chronic disease [8,9].'

4. When referring to boxing (lines 17, 22), please clarify whether this is "non-contact boxing" and use consistent terminology throughout. This distinction is important given the nature of the intervention and population.

Author response: Thank you for noting the inconsistencies. Both intervention arms involve non-contact boxing. Terminology has been edited throughout the manuscript for consistency. A description of non-contact boxing has also been added (pg 11).

Pg 11: 'In particular, non-contact boxing offers promise as a modality to help survivors reconnect with their bodies and ground into the present. Non-contact boxing combines high intensity training and bodily awareness through techniques including stretching, skipping, shadowboxing, pad work, and bag work [43]. It is shown to foster an enhanced sense of empowerment and ability to locate healthy aggression in survivors [44], along with increases in agency, resilience, self-esteem and perceived physical ability [45].'

5. The discussion of peer-led approaches needs further justification. The idea that support can be delivered by someone without formal clinical training is an important one but requires clearer explanation and referencing (see also p.10, line 38).

Author response: This is an important consideration. A paragraph outlining the benefits and challenges associated with peer-led approaches has been added to the Background under subheading 'Trauma-Informed, Peer-Led Interventions' (pg 9).

Pg 9: 'Peer-led support programs fit within this framework to create environments that privilege mutual support and relational healing while reducing power imbalances inherent in traditional therapeutic relationships [24,25]. These survivor-centred approaches, facilitated by individuals with lived experience rather than mental health professionals, offer a promising

alternative that can enhance treatment acceptability and help to overcome traditional barriers to care [24]. Rather than positioning trained clinicians as agents of empowerment, peer support recognises survivors as central agents of their own recovery [26]. Although growing evidence supports peer-led approaches for survivors, implementation challenges have hindered their adoption [27]. Thus, scaling peer-led interventions requires systematic evaluation of both effectiveness and implementation outcomes. Essential implementation components include developing comprehensive training protocols and establishing accessible supervision structures that enable peer facilitators to deliver interventions safely (for both themselves and those they support) and effectively [28,29].'

6. It would be helpful to clarify whether "champions," "peer-led," and "survivor instructors" are synonymous or distinct roles.

Author response: The protocol has been edited to ensure the terminology "peer-facilitators" is used throughout. When we describe the intervention as peer-led, we mean to say the sessions are run by peer-facilitators. Champions are peer-facilitators in training. We have added a line on page 23 to clarify this.

Pg 23: 'Champions become formal peer facilitators once program requirements are complete.'

7. Provide clearer justification for the focus on peer-led delivery, the theoretical and practical value is implied but not explicitly stated.

Author response: We appreciate this important note. A paragraph on the benefits of peer-led program delivery has been added on page 9 under the subheading 'Trauma-Informed, Peer-Led Interventions', also included above in response to 'Background, Comment 5'.

8. A brief overview of the evidence gaps that the RCT will address is needed before the aims section. This will help contextualize the decision to compare the two intervention arms.

Author response: Thank you for the suggestion. We have restructured the 'Background' manuscript to more clearly emphasise the research gap we are addressing. We have added text to expressly state the gap in the literature on page 12.

Pg 12: 'In sum, current research suggests that a program that incorporates both a physical health component with psychological component may increase treatment benefits for trauma-exposed individuals [40,48]. However, the unique combination of expressive writing and non-contact boxing is yet to be evaluated. These components can be delivered in a group-based setting, conferring additional benefits such as decreasing stigma and shame, and enhancing social connection [49]. Critically, this combined approach is suited to peer delivery, and taking a peer-led approach may further increase the acceptability and accessibility of support programs [50].'

9. Consider whether the trial should be framed as a hybrid effectiveness-implementation design. The current wording suggests the aim is to "develop an implementation strategy," but there may be scope to more explicitly evaluate both implementation outcomes (e.g., fidelity) and participant-level outcomes (e.g., self-efficacy).

Author response: Thank you for noting the lack of clarity on our indicated trial type. We are indeed explicitly evaluating both implementation and participant-level outcomes. We have made edits to state that the trial has dual individual-level (participant, facilitator) effectiveness and implementation (individual-level, site-level) aims (pg 15).

Pg 15: 'Our use of a hybrid effectiveness-implementation trial design allows dual consideration of a primary effectiveness (individual-level) and primary implementation (individual level, site-level) outcomes [63], enabling formal evaluation of both potential benefits of the intervention for survivors, and the acceptability and feasibility of intervention delivery within established organisations (health services, charities) which support survivors.'

Pg 15: 'We aim to complete a hybrid effectiveness-implementation cluster-randomised control trial to both evaluate the impact of Left Write Hook on the self-efficacy of adult survivors of child sexual abuse and other gendered violence, and concurrently develop and evaluate the fidelity of a train-the-champion implementation strategy for delivery in new settings.'

Methods

1. The process for obtaining GP clearance is unclear. Would asking participants to provide a clearance letter from their GP introduce unnecessary barriers, especially for those with histories of trauma or limited access to health care? Could a validated screening tool (e.g., the ESSA Adult Pre-Exercise Screening Tool) offer a more inclusive and efficient option?

Author response: Thank you for noting the lack of clarity around the process for GP clearance. Further detail on this process has been added under the Participant Recruitment subheading on pages 17-18.

Pg 17-18: 'To be included in the trial, participants must be 18 years or older, female identifying or gender diverse, self-report a history of childhood sexual abuse or gender-based violence including family violence, and be willing to provide contact details for their GP to enable the research team to make the GP aware of participation and to coordinate support if needed during participation. For the purposes of this study, the term 'gender-based violence' is inclusive of any experience of sexual violence, or any physical, sexual, or emotional violence from an intimate partner or family member, in either adulthood or childhood. Exclusion criteria are high levels of suicidality in need of more crisis management, evaluated by a clinical psychologist and informed by the Suicidal Ideation Attributes Scale [66]. All participants complete an intake interview with a clinical psychologist trained in risk formulation to collaboratively decide whether to participate. During the interview, the clinical

psychologist queries physical health, mental health and situational factors (e.g. ongoing violence) that may impact an individual's safe participation. Safe participation means that involvement in the program does not negatively impact an individual's physical or mental health, does not place them or others at risk of harm, and does not interfere with ongoing evidence-based treatments (e.g. participants undergoing EMDR may choose to delay participation until treatment is complete to avoid potential interference from the expressive writing component of Left Write Hook with memory reprocessing). Where safety concerns are identified, the clinical psychologist may recommend that the individual delay participation until their circumstances are more stable; for example, once an injury is healed, or external supports are in place. We initially pre-registered inclusion criteria to include a requirement for a letter of support from the GP, however decided to remove this to enhance equity of participation (i.e. in situations where the GP has been non-responsive). Instead, the participant will provide contact information for their GP and any other mental health professionals, with consent sought for them to be contacted if safety concerns arise in the intake interview or during the program. As per our risk management protocol (see Supplementary Materials for full protocol), if the trial team identify safety concerns, the GP or other health professionals will be contacted to confirm that the participant can take part safely and to ensure continuity of care. Where the GP or other health professionals involved in the person's care express concerns about safety of participation, this will be discussed with the individual so that they are aware of possible risks related to their involvement, and they will be invited to participate once their professional supports advise it is safe to do so.'

While we have sought to prioritise inclusivity throughout the trial design, it is extremely important that we take steps to ensure the physical and psychological safety of participants who enrol in the trial. The intake phone call helps to establish a connection and a sense of safety for participants, in line with principles of trauma-informed care. It also enables a more comprehensive assessment than a screening tool, offering insights into mental health, physical health and ongoing supports available during participation. We do not request a clearance letter from all participants, only in instances where risk to physical or psychological health are disclosed during the intake call. This not only helps us to verify that an individual will not be at risk of harm by participating in the trial, but also ensures we know support is available for this individual should they need it during the course of their participation. Left Write Hook is designed as an adjunct intervention and as this is a research trial, we are unable to support participants outside of each session. In light of this, we believe it is important to establish that individuals have access to external support before enrolling in the trial.

2. Clarify whether a "history of abuse" includes current experiences of abuse, this has ethical and safety implications for inclusion and risk management.

Author response: Thank you for raising this important response. We have added further detail on our intake process to clarify how we manage risk in situations of current abuse (pg 17).

Pg 17: 'During the interview, the clinical psychologist queries physical health, mental health and situational factors (e.g. ongoing violence) that may impact an individual's safe participation. Safe participation means that involvement in the program does not negatively impact an individual's physical or mental health, does not place them or others at risk, and does not interfere with ongoing evidence-based treatments...If a risk is identified, the clinical psychologist may recommend that the individual delay participation until their circumstances are more stable; for example, once an injury is healed, or external supports are in place.'

3. The term "safely participate" (p.13, line 55) could be unpacked. If this refers to psychological safety rather than physical risk, it would be helpful to clarify how the intervention delivery fosters a trauma- and violence-informed sense of safety. Consider describing specific mechanisms or processes that contribute to this (e.g., control of space, consent practices, facilitator behaviour).

Author response: Safe participation means that involvement in the program does not negatively impact an individual's physical or mental health, does not place them or others at risk, and does not interfere with ongoing evidence-based treatments. Additional details have been added to the method to clarify this under the heading 'Participant Recruitment' (pg 17, and included in response to 'Methods, Comment 2' above). We have also included a table outlining trauma-informed considerations embedded in both arms of the trial that aid prioritisation of participant safety throughout their involvement in the trial. Please refer to Table 2, page 21.

4. In the intervention description (p.15), it is unclear if the boxing delivered in the control arm differs in key ways (e.g., peer-led, empowerment focus). If so, this could confound interpretation, is any observed effect due to expressive writing or to the peer-led model?

Author response: Thank you for outlining this lack of clarity. We have added some more detail to outline how the intervention and control arms differ on page 23 under the heading 'Active Control – Boxing Only', also included below.

Pg 23: 'Boxing was selected as the exercise modality based on consumer input that identified it as the most acceptable exercise option for an active control, particularly in the context of consenting to randomisation. The control group therefore represents trauma-informed exercise options as usual, without three unique components of the Left Write Hook intervention: the peer-led nature of intervention; expressive writing; and the focus on empowerment that links from the expressive writing to the trauma-informed non-contact boxing component to encourage expression through physical movement.'

We have also noted in the Discussion (pg 36) that a future trial will need to isolate the active therapeutic components of Left Write Hook, if the trial indicates efficacy.

Pg 36: 'A mechanistic approach may additionally be taken in the future to unpack the active ingredients of Left Write Hook, notably, the compounded effects of peer-delivery and the empowerment focus.

5. Please specify which hormone(s) (e.g., cortisol) are being assessed.

Author response: The stress hormones we are testing are arachidonoyl, ethanolamide, 2-arachidonoyl glycerol, oleoylethanolamide, palmitoylethanolamide, stearoylethanolamide, lineoylethanolamide, arachidonic acid, cortisol, cortisone dehydropiandrosterone, dehydropiandrosterone-sulfate, progesterone, testosterone. As this is quite an extensive list, have provided a reference to Table S1 in the supplementary materials (pg 28 for reference, pg 1-5 for Table S1 in the supplementary materials), where a list of the specific stress hormones accessed by the reader, instead of listing each of the specific hormones in text. Please note that the BMJ Open editorial staff have requested our table of outcome measures be moved to the supplementary materials due to its length.

Pg 26. 'Hair samples indexing stress hormones (see Table S1 in supplementary materials for details) will be collected by the program facilitator, trained in collection procedure by a member of the research team'.

6. Trauma- and violence-informed principles are described as underpinning the intervention, but this needs clearer articulation. A table or diagram outlining how these principles are embedded in each arm would improve clarity.

Author response: This is a fantastic suggestion. A table outlining how trauma-informed principles are included throughout the trial has been included. Please see Table 2 on page 21.

7. The movement behaviour assessment (Table 2) requires a reference to support its reliability and validity.

Author response: The movement behaviours assessment is a bespoke single-item (4-part) question that has shown good validity against an activPAL criterion. Criterion validity against activPAL has been added in a note in Table S1 in the supplementary materials (pg 1-5, supplementary materials). Our measure captures similar concepts to one published after our study was preregistered that demonstrated good reliability and validity (Rodrigues et al., 2024; https://doi.org/10.1186/s12966-024-01667-7).

Pg 40, Table 3: '*Note.* *Criterion validity against activPAL (r = .35 sedentary, r = .40 standing, r = .58 moderate to vigorous intensity movement).'

8. Consider including the risk management protocol as an appendix, given the vulnerability of the target group.

Author response: Risk management protocol has been added as an appendix as suggested. Reference to this has been added to the manuscript (pg 18, pg 32).

Pg 18: 'As per our risk management protocol (see supplementary materials for full protocol), if the trial team identify safety concerns, the GP or other health professionals will be contacted to confirm that the participant can take part safely and to ensure continuity of care.'

Pg 32: 'The CI team includes clinical psychologists who are experienced in this subject area and will manage any risk in accordance with the Clinical Risk Management Protocol (provided in supplementary material).'

9. Clarify your approach to positionality. Given the survivor-led and co-designed nature of the intervention, it would be appropriate to reflect briefly on the research team's perspectives and positionalities (p.18).

Author response: Inclusion of a positionality statement is an excellent suggestion. This has been added to the method on page 25.

Pg 25: 'Our core research team is made up of predominately white, cisgender women, including clinical psychologists, researchers, and experts by lived experience. Some team members draw on both lived and learned expertise, though their primary roles in this study are grounded in academic and clinical perspectives. We acknowledge that these shared and divergent positionalities shape our approach to research, including study design, data collection, participant engagement, and interpretation of findings.'

We have also supplemented the section of the manuscript on Patient and Public Involvement (pg 25) to reflect on how our collaborative work with an advisory group complements and enriches the positionalities represented within the core research team.

Pg 25: 'This partnership supports critical engagement with dimensions of experience not wholly reflected in the core research team, particularly related to power, race and representation.'

10. The advisory group is comprised of CSA survivors, but the intervention appears broader in scope. Would it be appropriate to include survivors of other forms of gendered violence?

Author response: This is a great point. We do not ask our advisory group to disclose their trauma histories; however, we are aware that individuals within the group hold diverse experiences under the umbrella of gender-based violence, including experiences of child sexual abuse, adult sexual abuse, domestic violence and emotional abuse. We have edited the manuscript to broaden our language from 'sexual violence' and 'child sexual abuse' to 'gender-based violence' (pg 25).

Pg 25: 'With a focus on the co-production of research, this trial has been designed to actively involve and empower adult survivors of gender-based violence. The intervention has been designed and is delivered by survivors. Moreover, the trial has been designed to build the capacity of consumers to take ownership of the Left Write Hook intervention and to use their experiences to support others. In addition to the Lived Experience co-lead, the research has been co-designed by a paid Lived Experience Advisory Group (LEAG), comprising eight

individuals who have experienced gender-based violence, including both those who have and have not participated in the Left Write Hook program.'

11. Page 22, line 31: Please elaborate on the qualitative analysis approach. Thematic analysis is mentioned, but a brief description or reference (e.g., Reflexive Thematic Analysis) would enhance transparency.

Author response: We have added a reference to Braun and Clarke's 2006 paper on thematic analysis to contextualise our use of Reflexive Thematic Analysis (pg 32).

Pg 32: 'To determine implementation outcomes, qualitative data from semi-structured interviews with participants, champions, and peer-facilitators will be analysed using NVivo, in line with Reflexive Thematic Analysis [76].

Discussion

1. The case for peer-led delivery (p.27) needs to be better established earlier in the paper. Also, while the model may be low-cost, consider the implications of equitable pay for peer facilitators. The term "peer-led" should be clearly defined. Are peer facilitators also survivors? What support structures are in place?

Author response: Evidence for the benefit of peer-led delivery has now been added to the background section (pg 10, and copied above in response to Background Comment 5) and more detail about support for peer-facilitators has been added to the method (pg 24, under the subheading 'Facilitator and Participant Support'). Peer facilitators are peers due to their self-identification as survivors. All peer facilitators are paid in line with industry rates for casual facilitators of exercise-based interventions. Clarification on this point is provided on pg 20, and below.

Pg 20: 'Sessions are led by two paid peer facilitators: a Lead Facilitator, and assisted by an Assistant Facilitator, both of whom are survivors themselves. All peer facilitators are paid in line with industry rates for casually employed facilitators of exercise-based interventions'

Pg 24: 'Facilitators of both the Left Write Hook and boxing-only programs are supported by fortnightly group supervision sessions with a clinical psychologist. The clinical psychologist is on call for facilitator and participant support during and after all sessions for both the intervention and control groups. They also offer debriefs to both facilitators and participants via phone call or Zoom on an 'as needed' basis. Left Write Hook facilitators additionally have access to a community of practice, facilitated through WhatsApp by the Left Write Hook charity. This community of practice is not monitored by the research team.'

2. The term "trauma processing" (p.27, line 22) may not be accurate when describing sharing trauma narratives in group settings. Consider clarifying whether this is about expression, validation, or meaning-making.

Author response: The term "trauma-processing" has been replaced by "expression and validation". Evidence suggests that peer-led group interventions can support survivors of

sexual trauma to find their voices and express their emotions, and can decrease stigma and shame through validation from group members (pg 34).

Pg 34: 'Peer-led interventions in particular hold considerable promise for increasing self-efficacy of survivors and yielding mental health benefits via helping survivors to re-organize their sense of self, encouraging stabilisation (i.e., increased tolerance of difficult feelings) (24), expression and validation (i.e., sharing trauma narratives)(33,34,38), and re-integration (i.e., connection with others) (24).'

3. The support structures for peer facilitators (mentioned p.28, line 38) are not well described in the methods. If available, this detail should be included.

Author response: Detail has been added to the method (pg 24, under the subheading 'Facilitator and Participant Support', copied above in response to 'Discussion Comment 1').

VERSION 2 - REVIEW

**Reviewer**	**1**
**Name**	**Pebole, Michelle M.**
**Affiliation**	**Veterans Affairs Boston Healthcare System**
**Date**	**07-Aug-2025**
**COI**	

Thanks to the authors for their thoughtful revisions.

I have two remaining points for the authors to consider.

1) the authors should consider defining GBV in the introduction rather than the methods, given they use the term throughout the manuscript. additionally, authors should be consistent in the terminology used throughout the manuscript - this is really important for the field

2) I believe the authors should still provide more context for the bottom - up nature of this study (e.g., the intervention is built before it was tested), given that there is no information on evidence-based program development for this particular intervention (rather it seems this intervention was developed by integrating evidence from other interventions, not with input from groups of survivors / facilitators). This is still not clear in the descriptions, and I believe is important context. For example, the authors write that "In our pilot study with adult survivors of child sexual abuse..." yet, pilot studies are meant to focus primarily on feasibility / acceptability of a new program, not outcomes. In this case, it is my understanding the program was already being run at cost in the community, so it was not really a new intervention. Altogether, it is unclear how to make sense of how the program

was developed. Direct statements and transparency would be helpful for others working in this field / area.

Additionally, can the authors provide more direct context for the writing? It is my understanding no survivor needed to write about their past trauma, but it is unclear as written, and confusing as some may think this is a trauma focused writing prompt.

**Reviewer**	**2**
**Name**	**Rosenbaum, Simon**
**Affiliation**	**University of New South Wales**
**Date**	**12-Aug-2025**
**COI**	

Thanks to the authors for comprehensively addressing all of the comments. This manuscript makes a strong contribution to the existing literature and I look forward to seeing the results of the trial! Good luck!

I have some very minor suggestions for the authors and editor to consider but I do not need to see another version and already endorse this manuscript being published.

- under heading 'Trauma-Informed, peer-led interventions: consider adding a citation after first sentence ending in '...existing clinician- delivered therapies'

- under heading 'Body-Based Interventions': consider specifying and citing statement "there are a number of body-based therapeutic techniques..."

- likewise citation needed for statement about interventions reducing risk of chronic disease by up to 80%

- under heading "The Current Study": suggest toning down 'akin to type of current evidence-based programs survivors can easily access in the community'. Could be a counter argument that indeed appropriate services are not easily accessible. Consider just deleting word "easily"

VERSION 2 - AUTHOR RESPONSE

Reviewer: 1
Dr. Michelle M. Pebole, Veterans Affairs Boston Healthcare System
Comments to the Author:
Thanks to the authors for their thoughtful revisions.
I have two remaining points for the authors to consider.

1) the authors should consider defining GBV in the introduction rather than the methods, given

they use the term throughout the manuscript. additionally, authors should be consistent in the terminology used throughout the manuscript - this is really important for the field

We have now added a definition of gender-based violence in the introduction (pg 6). Additionally, we have made further edits to ensure that terminology is used consistently throughout the manuscript. We have changed reference to ‘gendered violence’ to instead be ‘gender-based violence’ and changed reference to ‘childhood sexual abuse’ to ‘child sexual abuse’ for consistency.

Pg 6: ‘Gender-based violence includes intimate partner violence, sexual violence and other harmful acts directed at individuals on the basis of their gender [3].’

2) I believe the authors should still provide more context for the bottom - up nature of this study (e.g., the intervention is built before it was tested), given that there is no information on evidence-based program development for this particular intervention (rather it seems this intervention was developed by integrating evidence from other interventions, not with input from groups of survivors / facilitators). This is still not clear in the descriptions, and I believe is important context. For example, the authors write that "In our pilot study with adult survivors of child sexual abuse..." yet, pilot studies are meant to focus primarily on feasibility / acceptability of a new program, not outcomes. In this case, it is my understanding the program was already being run at cost in the community, so it was not really a new intervention. Altogether, it is unclear how to make sense of how the program was developed. Direct statements and transparency would be helpful for others working in this field / area.

Thank you for noting the need to further clarify the origins of the Left Write Hook program. Additional detail on the bottom up nature has been added to the ‘Background’ section under the ‘Left Write Hook’ subheading for transparency around program development (pg 10-11) and the context around the pilot study (pg 11).

Pg 10-11: ‘Left Write Hook is a survivor-designed intervention, developed independently by a survivor of child sexual abuse in the community (i.e. rather than formally developed within a research context). The program was borne from the founder's own lived experience of gender-based violence and reflection on an unmet need for connection with others who shared similar experiences to her own . In considering how to foster a space in which survivors could come together as part of a shared journey towards healing, she recognized the profound impact of both creative practices (e.g. expressive writing, as opposed to written exposure techniques), and movement-based practices (e.g. boxing) in her own healing journey, and noted that such practices were often lacking from commonly offered psychological therapies (e.g., cognitive behavioural therapy, cognitive processing therapy, EMDR). Initial efforts included establishing a support group that came together to practice writing and boxing in a peer-led manner, and which received significant support within her local community. Subsequently, the Left Write Hook founder sought to formalise the program through the development of a manualized program, ultimately establishing a charity which is funded by philanthropic contributions to deliver the program across Victoria. Concurrently, the Left Write Hook charity sought to partner with researchers to formally establish the feasibility and acceptability of the intervention.’

Pg 11: ‘As noted earlier, prior research on the Left Write Hook has been conducted concurrently with the dissemination of the program within the local community within which it was established. After the program was developed, a pilot study was conducted with adult survivors of child sexual abuse to investigate the efficacy of the program and potential for future expansion [52].’

Additionally, can the authors provide more direct context for the writing? It is my understanding no survivor needed to write about their past trauma, but it is unclear as written, and confusing as some may think this is a trauma focused writing prompt.

We apologise for the lack of clarity. We have edited this section to clarify that there is no requirement that participants write about their past trauma, and that they may choose to write about any experience that comes to mind from the writing prompt (pg 19-20). Each writing prompt reflects themes from the session, as outlined in Table 1 (pg 19).

Pg 19-20: 'The first hour involves participants writing to recovery-focussed prompts designed to reclaim their life narrative and encourage meaning making (e.g., 'fighting back means...'), with the option of sharing with the group to encourage expression and validation. Writing prompts reflect the theme of each session. Participants are invited to write about anything that comes to mind for them in response to the writing prompts and session theme. While this means that they are not required to write about past trauma and may choose to write about any topic, participants do predominately choose to write about their experiences of trauma or its impact on them.'

Reviewer: 2
Dr. Simon Rosenbaum, University of New South Wales
Comments to the Author:
Thanks to the authors for comprehensively addressing all of the comments. This manuscript makes a strong contribution to the existing literature and I look forward to seeing the results of the trial! Good luck!

I have some very minor suggestions for the authors and editor to consider but I do not need to see another version and already endorse this manuscript being published.

- under heading 'Trauma-Informed, peer-led interventions: consider adding a citation after first sentence ending in '...existing clinician- delivered therapies'

Thank you for this suggestion. A citation has been added to support this claim.

- under heading 'Body-Based Interventions': consider specifying and citing statement "there are a number of body-based therapeutic techniques..."

Thank you for this suggestion. A citation has been added to support this claim.

- likewise citation needed for statement about interventions reducing risk of chronic disease by up to 80%

Thank you for noting this. A citation has been added to support this claim.

- under heading "The Current Study": suggest toning down 'akin to type of current evidence-based programs survivors can easily access in the community'. Could be a counter argument that indeed appropriate services are not easily accessible. Consider just deleting word "easily"

Thank you for noting this. We have removed the word ‘easily’ as per your suggestion.

Pg 14: ‘akin to the type of current evidence-based program survivors can easily access in the community.’

Reviewer: 1
If you have selected ‘Yes’ above, please provide details of any competing interests.: n/a

Reviewer: 2
If you have selected ‘Yes’ above, please provide details of any competing interests.: Not applicable

VERSION 3 - REVIEW

**Reviewer**	**1**
**Name**	**Pebole, Michelle M.**
**Affiliation**	**Veterans Affairs Boston Healthcare System**
**Date**	**14-Aug-2025**
**COI**	

Thank you to the authors for addressing these last suggestions. This study will add to the literature in this area, and I look forward to seeing how the trial progresses. Cheers!
